# Clinical genetic variation across Hispanic populations in the Mexican Biobank

**DOI:** 10.1038/s41591-025-04100-z

**Published:** 2026-01-21

**Authors:** Carmina Barberena-Jonas, Santiago G. Medina-Muñoz, Viankail Cedillo-Castelán, Tania Sepúlveda-Morales, Claudia Gonzaga-Jáuregui, ENSA Genomics Consortium, Viankail Cedillo-Castelán, Carlos Aguilar-Salinas, Carmina Barberena-Jonas, Sergio Canizales-Quintero, Luis Pablo Cruz-Hervert, Guadalupe Delgado-Sánchez, Elizabeth Ferreira-Guerrero, Leticia Ferreyra-Reyes, Cecilia Gutiérrez-López, Juan Eugenio Hernández-Avila, Alicia Huerta-Chagoya, Luis Juárez-Figueroa, Pablo Kuri-Morales, Eduardo Lazcano-Ponce, Carlos Magis-Rodríguez, Norma Mongua-Rodríguez, Hortensia Moreno-Macías, María de Jesús Ortega-Estrada, María José Palma-Martínez, Consuelo Dayzú Quinto-Cortés, Rosario Rodríguez-Guillén, María Luisa Ordóñez-Sánchez, Elsa Sarti-Gutiérrez, Karla Sandoval, Jaime Sepúlveda-Amor, Mashaal Sohail, Roberto Tapia-Conyer, María Teresa Tusié-Luna, José Luis Valdespino-Gómez, Norma Téllez-Vázquez, Oscar Velázquez-Monroy, Manuel Velázquez-Meza, Alexander G. Ioannidis, Lourdes García-García, Alexander G. Ioannidis, Andrés Moreno-Estrada

**Affiliations:** 1 Aging Research Center, Cinvestav Sede Sur, Center for Research and Advanced Studies of the National Polytechnic Institute, Mexico City, Mexico; 2 Posgrado en Ciencia e Ingeniería de la Computación, Universidad Nacional Autónoma de México, Mexico City, Mexico; 3 Laboratorio Internacional de Investigación sobre el Genoma Humano, Universidad Nacional Autónoma de México, Juriquilla, Mexico; 4 Instituto Nacional de Salud Pública (INSP), Cuernavaca, Mexico; 5 Department of Genetics, Stanford University, Stanford, CA USA; 6 Genomics Institute, University of California, Santa Cruz, Santa Cruz, CA USA; 7 Instituto Nacional de Ciencias Médicas y Nutrición Salvador Zubirán, Mexico City, Mexico; 8 Centro de Investigación y de Estudios Avanzados del Instituto Politécnico Nacional (Cinvestav), Irapuato, Mexico; 9 Secretaría de Salud, Mexico City, Mexico; 10 Present Address: Universidad Nacional Autónoma de México, Mexico City, Mexico; 11 Present Address: Broad Institute of MIT and Harvard, Harvard, MA USA; 12 Present Address: Tecnológico de Monterrey, Monterrey, Mexico; 13 Present Address: Universidad Autónoma Metropolitana, Mexico City, Mexico; 14 Present Address: University of California, San Francisco, San Francisco, CA USA; 15 Present Address: Fundación Carlos Slim, Mexico City, Mexico

**Keywords:** Risk factors, Pharmacogenomics, Personalized medicine, Medical genetics, Translational research

## Abstract

Genetic testing for specific alleles is often recommended based on an individual’s ancestry. However, the frequency of pathogenic and pharmacogenomic alleles across different Hispanic groups has not been well characterized, and existing guidelines often fail to recognize the geographic and ancestral diversity within these populations. Here analyzing data from 6,011 individuals from the nationwide Mexican Biobank, we show that Mexican individuals have striking regional differences in biomedically relevant allele frequencies, shaped both by their overall admixture proportions, but also by the local Indigenous ancestral groups contributing to their genome (for example, Nahua in central Mexico, Zapotec in the South or Maya in the Yucatan peninsula). We found ancestry-specific patterns with clinical implications that could not have been detected without a local ancestry-informed approach, including variants affecting fentanyl (rs2242480) and statin (rs4149056) metabolism, examples particularly relevant to the epidemiology of Hispanic populations. This analysis framework could inform genetic testing guidelines across the Americas. We are making available the results for 42,769 biomedically relevant genotyped variants through MexVar, a user-friendly platform designed to improve access to genomic data for the scientific community and support genetic analyses for populations of Mexican descent worldwide.

## Abstract (web summary)

Analyzing data of the Mexican Biobank project, a new study finds regional differences in clinically relevant genetic frequencies and presents MexVar, a publicly accessible resource designed to support ancestry-informed genetic testing.

## Main

Clinical recommendations often consider observed allele-frequency differences in specific populations, recognizing the need for tailored approaches in precision medicine^1,2^. For example, individuals of Ashkenazi Jewish descent are advised to undergo genetic testing for *BRCA1* and *BRCA2* pathogenic variants due to a higher prevalence in this group of these variants, which substantially increase the risk for breast and ovarian cancers^3–5^. Similarly, it is often recommended that Hispanic/Latinos be tested for specific genetic markers, such as those influencing drug metabolism^6^. In practice, the American College of Medical Genetics and Genomics (ACMG) in the United States recommends carrier screening if a clinically relevant variant reaches a frequency of 1:200 in a population^7^. However, how such a population is defined is not clear, and existing clinical guidelines often completely overlook differentiation among Hispanic/Latino populations, treating them as a single ethnicity. This is particularly relevant for Mexican individuals, whose genomes are often admixed with varying continental ancestry contributions due to historical migrations and also include contributions from different subcontinental regional ancestries, particularly distinct Indigenous American ones^8–11^.

**Extended Data Fig. 1:**
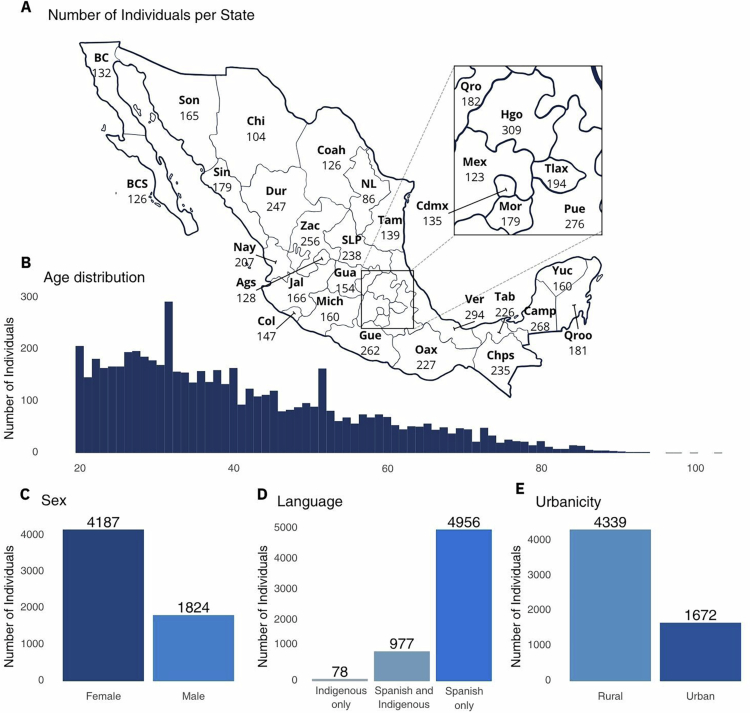
Geographic and demographic summary of the Mexican Biobank. (**a**) Number of individuals per state in the Mexican Biobank cohort. Inset maps show detailed views of central and southeastern states. (**b**) Age distribution. (**c**) Sex distribution. (**d**) Distribution by language spoken and (**e**) urbanicity.

To fully understand and explore the clinical genetic consequences of this diversity, a nationwide biobank that captures the unique genetic landscape of Mexicans is essential. The Mexican Biobank (MXB) provides an invaluable resource by including genetic data from individuals across 898 localities nationwide, in both urban and rural areas^11^. This biobank currently includes 6,011 individuals from all 32 states across Mexico (Extended Data Fig. 1), genotyped for 1.8 million variants on the Multi-Ethnic Global Array (MEGA), which provides extensive coverage of clinical variants and those associated with diseases and pharmacogenomics (PGx). The comprehensive design of the MXB enables in-depth characterization of genetic variation, making it a crucial tool for advancing precision medicine in the Mexican population (for further details on the inclusion criteria and genotyping array, see Methods).

Here we demonstrate, using the MXB, that genetic diversity within Mexicans is far more intricate than a monolithic ethnic label like ‘Hispanic’ or ‘Latino’ can capture. This complexity arises from two key factors. First, Mexicans exhibit varying proportions of Indigenous American, European and African ancestries, which substantially influence the frequency of biomedically relevant variants, such as those associated with pharmacogenetic traits. Second, there is substantial regional subcontinental differentiation within the Indigenous American source ancestries of Mexicans, stemming from pre-Columbian population genetic variation, from Arido-American groups in the north to Mesoamericans around the central plateau of Mexico City to southern Oaxaca and the Maya of the Yucatán Peninsula.

We have found that subcontinental variation among these Indigenous American subgroups contributing ancestry to admixed Mexicans can result in geographic allele frequency variation across Mexico that is as large as, or even larger than, that observed between many continental groupings worldwide, such as Europeans and East Asians.

To better explore these complexities, we developed MexVar, an interactive platform that integrates multiple datasets and provides a comprehensive tool for exploring the frequency variation of biomedically relevant variants in Mexico, accounting for geography and ancestry at fine scales. MexVar empowers researchers, clinicians and broader users by offering access to crucial genetic information that enables precision medicine strategies tailored to the diverse genetic backgrounds of Mexican and broader Hispanic/Latino populations.

## Results

### Biomedical variants are differentiated across geography and ancestries

**Extended Data Fig. 2:**
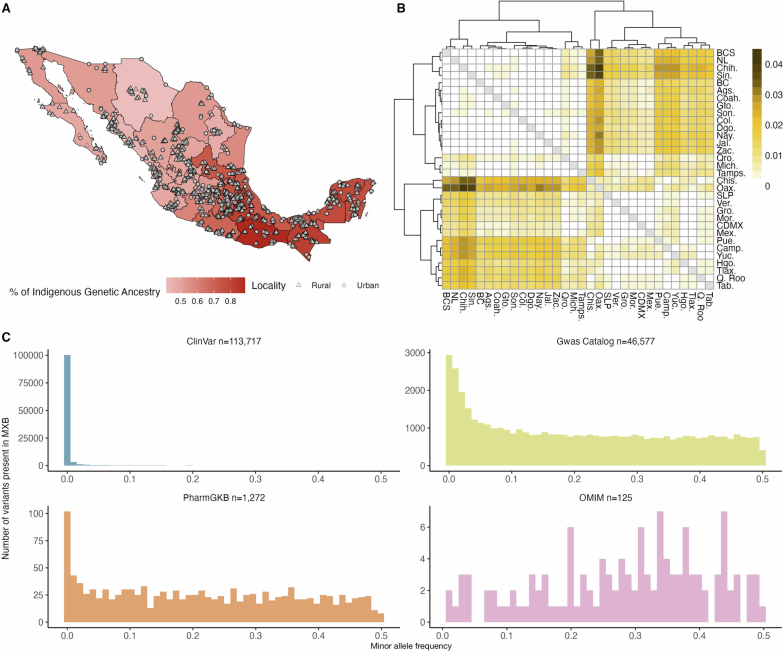
Allele frequency landscape of clinically relevant variants in the Mexican Biobank. (**a**) Geographic map of Mexico showing the sampling locations of the 6,011 individuals in the MXB. Each point represents a sample, with shape indicating urban (triangle) or rural (circle) origin. States are color-coded by average proportion of Indigenous genetic ancestry. Image in panel **a** is reproduced with permission from Valle-Jones D. (ref. ^38^), used under a free use license. (**b**) Pairwise Fst heatmap showing genetic differentiation between Mexican states based only on clinically relevant genetic variants. (**c**) Distribution of minor allele frequencies (MAFs) for clinically relevant SNPs in the MXB dataset, grouped by source database (ClinVar, GWAS Catalog, PharmGKB, OMIM).

First, we defined biomedically relevant variants as those established, or implicated, in disease risk, diagnosis or treatment response. To curate these, we leveraged multiple sources, including Pharmacogenomics Knowledge Base (PharmGKB), Online Mendelian Inheritance in Man (OMIM), ClinVar and the GWAS Catalog^12–15^. These aggregate data from large-scale genomic investigations, clinical case reports and association studies. We required our curated variants to be present in at least one of the databases and genotyped in the MXB (Supplementary Table 1). We then calculated the allele frequency for all curated variants in the databases (Extended Data Fig. 2c). This analysis shows that large differences exist in overall curated variant numbers and frequency distributions between the databases.

Examination of average continental ancestry proportions across Mexican states (Extended Data Fig. 2a) revealed heterogeneity, with southern states, particularly Oaxaca, showing a higher proportion of Indigenous American ancestry, while northern states exhibited a lower proportion. As expected and previously shown, European ancestry displayed an inverse relationship with Indigenous ancestry (Supplementary Fig. 1)^10,11^. This affects measures of genetic differentiation as shown next.

We calculated Fst values for biomedically relevant variants in a pairwise comparison between Mexican states, observing the largest genetic differentiation along a north-to-southeast gradient (Extended Data Fig. 2b). This is consistent with previous reports^11^ using all genetic variants, regardless of functional category, meaning that the frequencies of biomedically relevant variants appear to behave similarly to variants across the entire genome. We found that the highest Fst values were observed between southern states (that is, Oaxaca and Chiapas) and northern ones (that is, Chihuahua and Sinaloa). These also show the highest and lowest average Indigenous American ancestry, respectively, suggesting that part of the observed differentiation is driven by European-Indigenous admixture clines in modern Mexico.

**Fig. 1:**
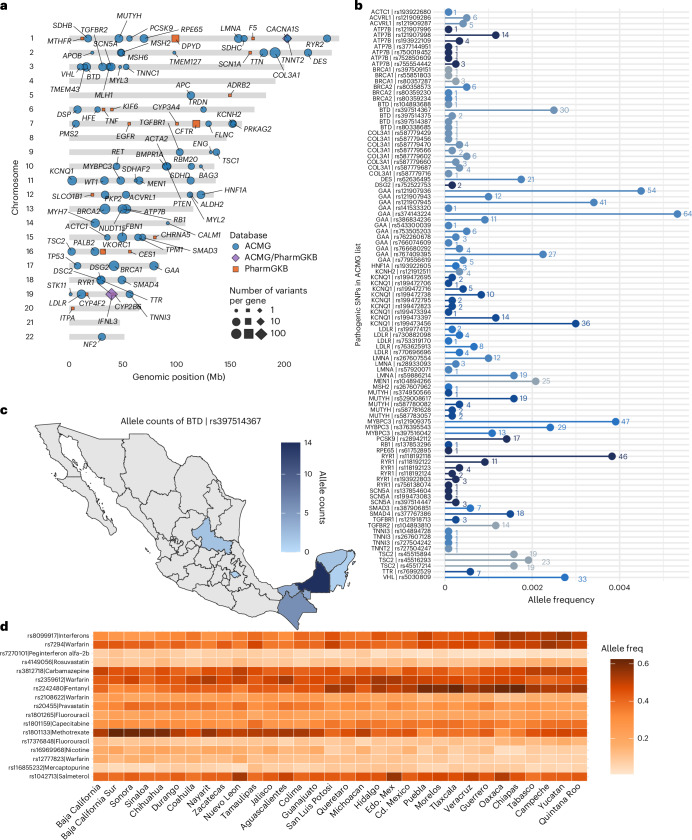
Distribution and allele frequency of clinically relevant and actionable variants across the genome and in the MXB. **a**, Genomic positions of pathogenic variants in ACMG SF v3.2-listed genes and PGx variants annotated in PharmGKB. Each dot represents a gene, colored by database source. Dot size reflects the number of variants genotyped in MXB, per gene. **b**, Nationwide allele frequencies of ACMG-listed pathogenic variants with nonzero frequency. Variants are grouped by gene. **c**, Geographic distribution of the pathogenic BTD variant rs39751467, showing higher allele counts in the Yucatán Peninsula. Image in panel **c** is adapted from ref. ^38^, used under a free use license. **d**, Heatmap of allele frequencies for pharmacogenomically relevant SNPs across states. Only variants with a MAF > 0.05 in the MXB were included. SNPs are annotated with their associated drugs according to PharmkGKB database, where multiple associations exist, the one with the highest evidence level was selected. Edo. Mex, Estado de México; Cd. Mexico, Ciudad de México; freq, frequency.

To explore these variants further, we defined a focused subset of clinically relevant and actionable variants by integrating PGx alleles from PharmGKB (levels 1A, 1B, 2A, 2B) and pathogenic or likely pathogenic variants from ClinVar occurring in ACMG Secondary Finding List (SF) v3.2-listed genes associated with cardiovascular, metabolic, hereditary cancer and other actionable conditions (Fig. 1).

Of the ClinVar-reported pathogenic and likely pathogenic variants in ACMG-listed genes, 1,057 sites were genotyped in the MEGA array, involving 73 of the 81 recommended genes. Among these, 99 variants in 33 genes had a nonzero minor allele frequency (MAF; Fig. 1b). Variants in *GAA*, *MYBPC3*, *RYR1*, *KCNQ1* and *BTD* genes showed particularly high frequencies. The variant rs39751467 in the *BTD* gene, for instance, displayed a striking geographic pattern, almost exclusively present in the Yucatán Peninsula (Fig. 1c). Pathogenic BTD variants are associated with biotinidase deficiency^16^ (MIM, 253260), but oral biotin supplementation is possible and, when patients are identified early enough, can prevent neurological damage.

**Fig. 2:**
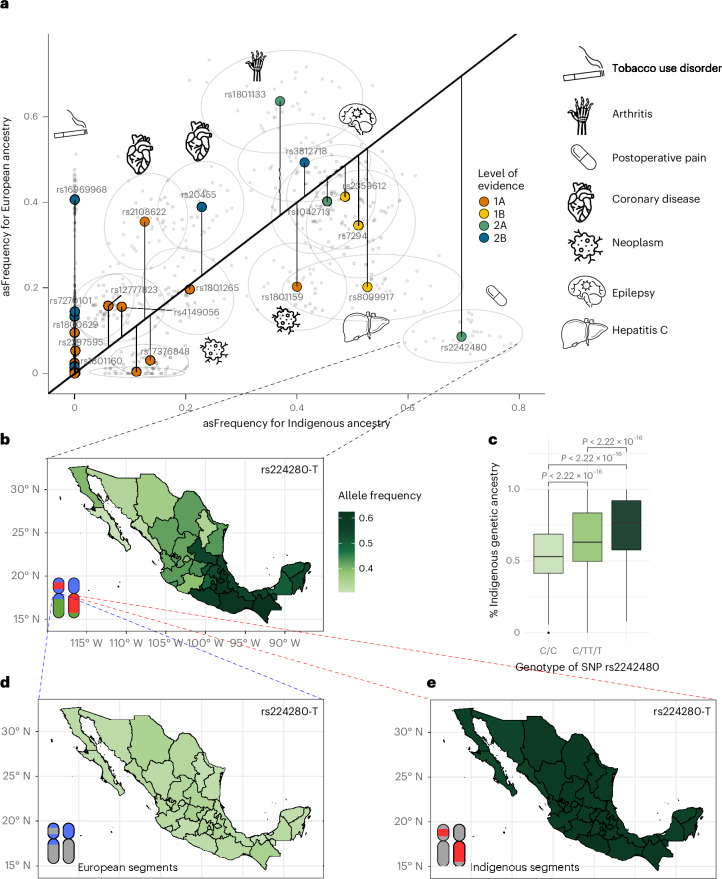
asF of PGx variants in the MXB. **a**, Comparison of asF for 58 high-evidence SNPs associated with drug response, curated from the PharmGKB database. Allele frequencies were calculated exclusively within chromosomal segments assigned to either Indigenous or European ancestry. Gray points represent state-level asF estimates, while colored points indicate the national average for each ancestry. Distance from the diagonal reflects the degree of ancestry enrichment. SNPs are color-coded by level of evidence, and icons indicate associated clinical phenotypes for selected variants. **b**, Geographic distribution of allele frequencies for SNP rs2242480-T, illustrating a regional gradient with higher frequencies in southeastern Mexico. **c**, Proportion of Indigenous ancestry among individuals with different genotypes at rs2242480, showing increased Indigenous ancestry in individuals carrying the C allele. The centerline represents the median, the box bounds correspond to the 25th and 75th percentiles, and whiskers extend to the most extreme values within 1.5× the interquartile range (*n* = 6,011, *t* test two-sided). **d**,**e**, Distribution of asF for rs2242480-T, showing enrichment of the C allele within Indigenous chromosomal segments (**e**) compared to European ones (**d**), highlighting population-level genetic differentiation relevant to PGxs. Images in **b**, **d** and **e** are adapted from ref. ^38^, used under a free use license.

**Fig. 3:**
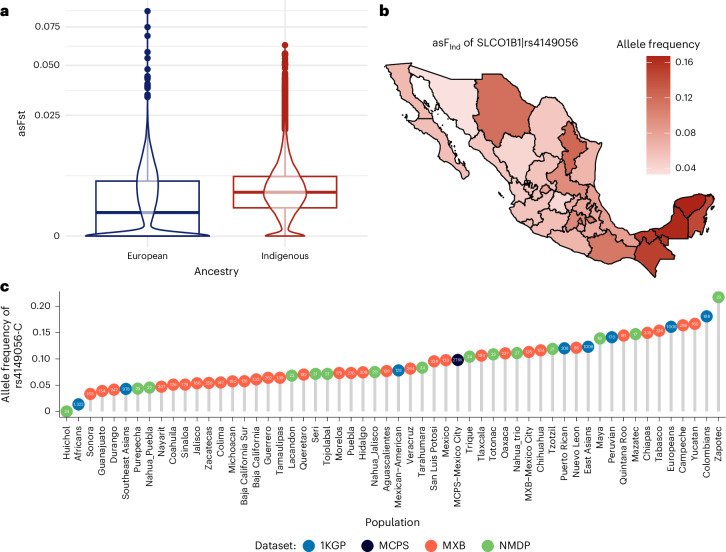
Greater differentiation is observed across Indigenous segments than across global populations. **a**, Boxplots indicate the distribution of asFst values using biomedically relevant SNPs only for genomic segments of Indigenous and European ancestry in the MXB: the centerline represents the median, the box bounds correspond to the 25th and 75th percentiles, and whiskers extend to the most extreme values within 1.5× the interquartile range; points beyond this are outliers. The violin plots display the kernel density estimation of the data distribution. A significant difference is observed (*n* = 42,769, *P* = 0.042, 95% confidence interval = −1.314061 × 10^−4^ to −2.337399 × 10^−6^, *t* test two-sided), with Indigenous segments showing higher asFst values, indicating greater genetic differentiation within this ancestral component. **b**, Geographic distribution of asF for the C allele of rs4149056 in Indigenous genomic segments across Mexico. The map reveals regional variation in the ancestral Indigenous American substratum of Mexicans, with much higher frequencies in the ancestrally Mayan Yucatan. Image in **b** is adapted from ref. ^38^, used under a free use license. **c**, Bar plot showing the frequency of rs4149056 (C allele) in Indigenous American segments (asF) across MXB states compared with the frequency in Indigenous Mexican populations from the Native Mexican Diversity Project (NMDP) shows a correspondence in between each state and the local Indigenous groups. Frequencies for worldwide groups from the 1000 Genomes Project (1KGP) and the MCPS are provided for comparison. The sample size for each population is inside each dot.

We then focused on PGx alleles totaling 58 SNPs and 22 genes (Supplementary Table 2), we obtained allele frequencies across each state in Mexico (Supplementary Fig. 2) and even within this single nation, observed substantial heterogeneity across states (Fig. 1d). For example, rs2242480—associated with fentanyl metabolism^17^—showed a clear north-to-south gradient, while rs1801133—linked to methotrexate response—had higher frequencies in the north. In the following section, we aim to demonstrate that such patterns arise from two distinct sources that together have a key role in shaping clinical variation in Mexico: admixture gradients of continental ancestry (illustrated in Fig. 2) and geographic differentiation in Indigenous American ancestry sources across Mexico (highlighted in Fig. 3).

**Extended Data Fig. 3:**
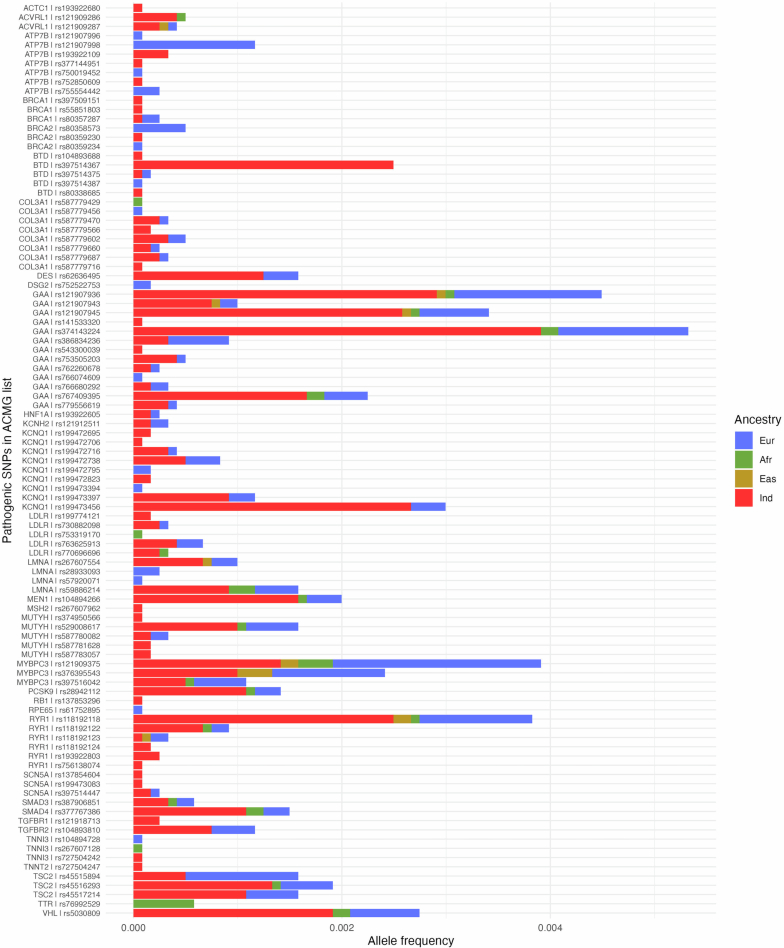
Allele frequencies of ACMG-listed pathogenic variants stratified by ancestry. Nationwide allele frequencies of pathogenic or likely pathogenic variants reported in ACMG SF v3.2 genes, identified in the Mexican Biobank. Only variants with nonzero frequency are shown. Bars are grouped by gene, and colors indicate the proportion of each allele frequency attributed to continental ancestries, as estimated through local ancestry inference.

To that end, we performed further analyses to dissect the allele frequencies of the clinically relevant and actionable variants into their ancestry-specific frequency components (Fig. 2 and Extended Data Fig. 3). Ancestry-specific frequencies (asF_*x*_) compute allele frequencies only for alleles observed in chromosomal segments inherited from the ancestry of interest. For example, since an admixed individual in Mexico might have some genomic segments of a particular chromosome inherited from European ancestors and others from Indigenous American ancestors, we would include the allele on that individual’s chromosome in the Indigenous American-specific frequency estimate only if it lays within a segment inherited in that individual on that chromosome from Indigenous American ancestors (for details, see ‘Local ancestry analysis’).

In our asF_*x*_ analysis, we compared the distribution of all 58 pharmacogenetic variants across Indigenous, European and African ancestry genomic segments. Of these, only 32 variants were found in all three ancestries (Supplementary Table 3). Among these, most showed marked deviations in pairwise comparisons, indicating clear enrichment toward a specific ancestry. For example, 19 variants were enriched in European ancestry segments when compared to Indigenous segments, and 20 compared to African segments (Supplementary Table 3). Among the 19 variants enriched in European segments versus Indigenous segments, 12 were either minimally represented or entirely absent in Indigenous ancestral segments of the MXB population (Fig. 2a).

We identified nine variants enriched for Indigenous ancestry, of which rs2242480-T (CYP3A4*1G; Fig. 2b) showed the highest Indigenous enrichment. Its allele frequency distribution exhibits a north-to-south increase within Mexico (Fig. 2b), and the higher frequency of the T allele correlates with higher Indigenous genetic ancestry proportions (Fig. 2c and Supplementary Fig. 4). This is consistent with the previously observed European ancestry cline in Mexico, and the allele-specific frequency asF_*x*_ pattern, which shows that ancestry-specific allele frequency (asF) in individuals with genomic segments of European ancestry (asF_Eur_) has a lower frequency of the effect allele for rs2242480 compared to the asF observed in individuals with genomic segments of Indigenous American ancestry (asF_Ind_; Fig. 2d,e and Supplementary Fig. 5). Moreover, in the Indigenous segments, this genetic variant has a consistent allele frequency of 60% across states. When looking at the African segments (asF_Afr_; Supplementary Figs. 3–5), we found that the frequency of the effect allele was even higher than in Indigenous segments. However, the overall contribution of African segments to the MXB samples is considerably smaller than that of Indigenous segments. As a result, while both ancestries contribute to the overall frequency of the variant in the population of Mexico, Indigenous ancestry has a more prominent role in shaping the observed nationwide distribution, which reflects regional differences in continental ancestry proportions.

Another African-enriched variant is rs1801265 (DPYD*9a), where genotypes AA + AG are associated with reduced drug toxicity when treated with capecitabine or fluorouracil in individuals with colorectal neoplasms, compared to genotype GG. This variant shows a frequency of 42% in African segments, compared to 21% and 20% in Indigenous and European segments, respectively (Supplementary Figs. 3–6 and Supplementary Table 3).

### Subcontinental variation within the Indigenous American ancestry of Mexicans can be larger than between other ancestries worldwide

We then compared the distribution of ancestry-specific Fst (asFst) values across all biomedically relevant variants between Mexican states for Indigenous American and European genome segments. We identified a significant difference (*P* = 0.042), where Indigenous American segments exhibit higher mean Fst across states than European segments, reflecting greater geographic differentiation in allele frequencies across Mexico in this ancestry component. European chromosomal segments predominantly show low Fst values, likely due to a common Spanish origin across the nation, albeit with some extreme asFst alleles possibly arising from colonial founder effects (Fig. 3a and Supplementary Fig. 7).

To illustrate this greater frequency differentiation in Indigenous American ancestry segments, and to highlight that regional frequency differences do not stem only from differential admixture but rather from ancestral variation in the Indigenous American substratum of Mexico, we considered one of the best documented pharmacogenetic variants—rs4149056 (c.521T > C found in SLCO1B1*5 and SLCO1B1*15). This SNP has been strongly associated with the response to several statins (Supplementary Fig. 8), such as atorvastatin, pravastatin and simvastatin, in individuals with hyperlipidemia. When controlling for admixture by considering each ancestry separately, we found that although the variant is overall more frequent in European haplotypes, the effect allele frequencies in Indigenous American segments show strong geographic differentiation, being much higher in the southeast than in central-northern Mexico (17% in Yucatan versus 3% in Sonora). This extreme regional frequency variation in the Indigenous American ancestry component of Mexicans is larger than the differences in frequency for this allele between continental populations worldwide (ranging from 5% in Southeast Asians to 16% in Europeans; Fig. 3). Variation within the Indigenous component is what also drove geographic variation in the previously mentioned BTD variant (rs39751467), which is nearly exclusive to Indigenous segments from the ancestrally Mayan Yucatan.

To investigate the driver of this regional frequency difference in the Indigenous ancestry component, in Mexicans, we explored the frequencies of rs4149056 in another cohort, the Native Mexican Diversity Panel (*n* = 454)^10^. Although having a smaller sample size compared to the MXB dataset, this dataset includes a wide range of Indigenous groups with self-identified ethnicity (and >90% Indigenous ancestry). In this independent cohort, we also observed a higher allele frequency in the southeast (with Zapotec having over 22% frequency) versus the west (with Purepecha having 4% and Huichol having 0%), paralleling the regional differences we observe in the MXB (Fig. 3c and Supplementary Figs. 8 and 9). This suggests that pre-Columbian native population structure still shapes the frequencies of pharmacogenetic variants in Mexicans, resulting in substantial regional differences across the MXB.

Subcontinental variation of pharmacoalleles is not restricted to just the Indigenous American component. For SCN1A rs3812718-T, where TT carriers require higher carbamazepine doses than CC carriers, the allele distribution is heterogeneous across both asF_Ind_ and asF_Eur_ (Supplementary Fig. 10).

### Factors impacting clinical genetic variation in Mexico

**Fig. 4:**
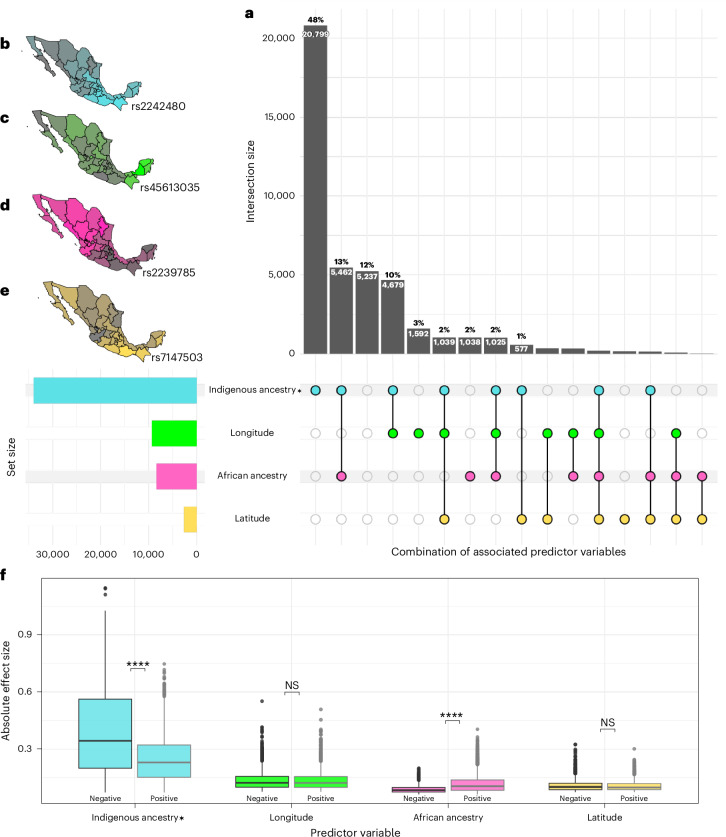
Geographic and ancestry-related contributions to genetic variation in the MXB. **a**, UpSet plot summarizing results from a GLM assessing the association of genetic ancestry (Indigenous, African) and geographic variables (latitude, longitude) with the distribution of 42,769 clinically relevant variants. Vertical bars represent the number of SNPs significantly associated with each term or combination of terms (*P* < 0.05); horizontal bars indicate the total number of variants associated with each variable. Combinations accounting for <1% of variants are not labeled. **b**–**e**, Geographic distribution of allele frequencies for four representative SNPs in the MXB, each illustrating a distinct pattern of genetic variation. Star indicates that the Indigenous ancestry component can be interpreted as a mirror of European ancestry in the opposite direction, due to their negative correlation. **b**, rs2242480 (fentanyl metabolism), enriched in individuals with higher Indigenous ancestry. **c**, rs45613035 (asthma risk), showing a longitudinal gradient with elevated frequencies in eastern Mexico. **d**, rs2239785 (apolipoprotein L1, kidney disease), associated with African ancestry. **e**, rs7147503 (body mass index), exhibiting a latitudinal gradient with increased frequency in the south. Images in **b**–**e** are reproduced with permission from Valle-Jones D. (ref. ^38^), used under a free use license. **f**, Boxplots of the absolute effect size from the GLM for each variable, stratified by direction (positive versus negative effect). The Indigenous ancestry term shows significantly stronger negative effects compared to positive ones (*t* test two-sided, ****P ≤ 0.001; NS, not significant, *P* > 0.05. Indigenous ancestry, *t*(33,887) = 79.1, *P* < 1 × 10^−300^; longitude, *t*(9,316) = 0.99, *P* = 0.323; African ancestry, *t*(6,888) = –36.9, *P* = 6.9 × 10^−272^; latitude, *t*(2,588) = 1.92, *P* = 0.055), suggesting a systematic bias in allele frequency patterns toward European enrichment.

To characterize and quantify the underlying factors contributing to the wide frequency variation, we applied a generalized linear model (GLM) to all biomedically relevant variants with a MAF 5% (42,769), incorporating geographic variables (latitude and longitude) and continental genetic ancestry as predictors. The results revealed that 62% of the variants (*n* = 26,517) show a significant association with the Indigenous American ancestry proportion (*P* < 0.05; Fig. 4). Geographic variables predominantly showed an effect when combined with ancestry components (Indigenous or African), and <6% (*n* = 2,465) of the variation is solely explained by geography (Fig. 4a). We also identified 12% (*n* = 5,237) of variants where variation cannot be attributed to any of the variables we modeled, indicating a more complex scenario. To illustrate these findings, we selected four representative SNPs, each highlighting a different predictive factor from the GLM analysis (Fig. 4b–e). We then examined the estimated effect of each variable. For genetic ancestry, most variants had a negative effect within the Indigenous American component (Fig. 4f, *P* ≤ 0.001). Accordingly, this means that European ancestry was associated with a higher effect on allele frequencies, suggesting a strong selection bias in the clinical variants ascertained in these databases, with most deriving from studies on European-descent individuals. We observed a similar, significant difference between positive and negative effects for African ancestry. There was no significant difference between longitude and latitude regarding positive and negative effects. To further investigate patterns of genetic differentiation, we calculated the absolute difference in allele frequency between ancestries (Supplementary Fig. 12) and expanded on these findings in Supplementary Fig. 13 to reveal genome-wide patterns of allele frequency differences, with most variants showing enrichment towards European ancestry. We found that this pattern was consistent across databases, further supporting a discovery bias rooted in European-centric studies.

Other clinically relevant variants did not show geographically structured patterns but segregated exclusively in genomic segments of African ancestry, which also deserves particular attention. This ancestral component is less prevalent than the Indigenous or European ancestries in the MXB, and its distribution is more uniform across Mexico. The vast majority of MXB individuals have 1–5% African ancestry nationwide (Supplementary Fig. 11). High-frequency variants in African source populations may have been inherited via founder effects during the transatlantic slave trade and maintained in the African component of admixed Mexicans. This appears to be the case for a variant in the *APOL1* gene (rs73885319-G from ClinVar and GWAS Catalog), where all occurrences of the risk allele (*n* = 68) were observed in African haplotypes, supporting the hypothesis of an African origin of this MXB allele, which has been strongly associated with end-stage kidney disease, early-onset hypertension and cardiovascular disease^18^. Elevated frequencies of the *APOL1* risk allele have been found in African-American, sub-Saharan African and Western African populations. Recent studies have found elevated frequencies outside Africa and the United States, including Caribbean and Central American populations with high proportions of African ancestry^19^. In our analysis, the allele was not observed at higher frequencies among individuals with greater African ancestry.

### MexVar is an interactive database to explore biomedically relevant variants in the context of ancestry and geography

**Fig. 5:**
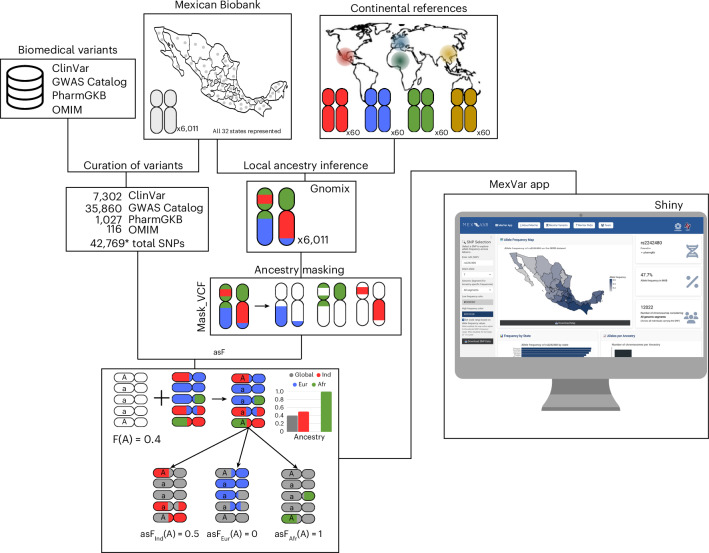
MexVar is an interactive platform for exploring ancestry- and geography-informed allele frequencies in the MXB. MexVar integrates genomic and ancestry data from 6,011 individuals in the MXB, using continental reference panels to perform local ancestry inference. A curated set of 42,769 unique SNPs, compiled from ClinVar, GWAS Catalog, PharmGKB and OMIM, was analyzed. Although 44,305 total entries were included across databases, some SNPs appear in more than one database. The pipeline includes local ancestry inferences, ancestry masking and the computation of asF. MexVar is built on a user-friendly Shiny interface that enables interactive visualization of geographic and ancestry-specific variant distributions across Mexico. The platform supports the interpretation of clinically relevant genetic variation in the context of population structure, advancing equitable precision medicine.

To date, there is no publicly available platform that allows for user-friendly consultation of allele frequencies at a nationwide scale in Mexico. To close this gap, we incorporated the allele frequencies estimated in the MXB (genome wide and ancestry specific) into a graphical user interface platform named MexVar (Fig. 5). This is an R-based interactive platform for end-users, allowing them to explore and visualize allele frequency data in real time with the option of incorporating a local ancestry analysis (ancestry specific) informed approach. The output includes a dynamic map of Mexico featuring overall and asF, a bar graph visualization, and detailed descriptions of variant effects. Users can also download variant frequency data for further analysis. Additionally, users have the flexibility to tailor the plot esthetics, such as including color schemes and titles, to suit their preferences. MexVar includes a dedicated ‘variant details’ tab with curated metadata, allowing users to access studies related to each allele. MexVar’s design allows for the integration of additional data as it becomes available, whether through increased sample size or through the inclusion of sequencing data from technologies such as whole-genome sequencing, exomes or long-read sequencing. Additionally, MexVar can incorporate newly reported variants from external databases. This scalability and adaptability make MexVar a long-term tool for genetic research and clinical applications. MexVar is available at https://morenolab.shinyapps.io/mexvar/.

## Discussion

Current clinical guidelines often fail to account for the genetic diversity within Hispanic/Latino populations, considering them as a single homogeneous label. This approach overlooks substantial regional genetic differences, particularly in highly diverse countries like Mexico, where Indigenous American ancestry source populations vary widely across the country. Our study addresses this gap by leveraging MXB data to characterize regional differences in allele frequencies of biomedically relevant variants across all 32 states of Mexico. Our results revealed several patterns of differentiation, including a North-South cline that aligns with previous findings on genetic diversity across Mexico using all autosomal variants^10,11^.

We found that 60% of biomedically relevant variants are influenced by continental ancestry proportions (admixture). These findings align with previous PGx studies in Latin America, where many variant frequencies correlate with continental genetic ancestry^20–23^. Most of the remaining variation is attributable to geographic genetic variation. We demonstrate that this stems largely from remarkable differences in allele frequencies within the Indigenous American ancestry substratum of Mexicans, driven by pre-Columbian Indigenous population substructure. Indeed, genetic differences among Indigenous source populations from different regions, such as the Nahua, Maya or Mixtec, correlate directly with geographic frequency differences of clinically relevant variants in the MXB cohort. Even if participants may not necessarily self-identify with a particular neighboring Indigenous ancestral group, these groups have contributed to their genetic ancestry and have thus shaped their genetic risk.

These results collectively suggest that using descriptors such as Latino or Hispanic to imply a genetically homogeneous group risks overlooking important PGx differences rooted in ancestral diversity. Finally, approximately 12% of the variants did not correlate significantly with either ancestry, geography or a combination of these factors, unveiling a more complex scenario that should be explored further. Despite the current bias in characterized variants, which have mostly been ascertained in European populations, we were able to discover some variants more common in Indigenous ancestry segments than in European. These are particularly relevant for local epidemiology, as current clinical guidelines dominated by European-derived frequency data may not recommend routine testing for them. rs2242480 (CYP3A4*1G) illustrates a striking case showing a positive correlation between the effect allele (T) and Indigenous genetic ancestry. In the MXB, the T allele is highly prevalent in southern states such as Chiapas (86% carriers, 38% homozygous), compared to northern states like Baja California Sur (53% carriers, 9% homozygous). The T allele is linked to reduced fentanyl metabolism and a lower dose requirement for women in postsurgery pain management, with implications for safer opioid dosing during childbirth^24,25^. While the United States faces an opioid crisis, with fentanyl as the leading cause of death among adults aged 18–45 years^26,27^, Mexico deals with opioid shortages that limit treatment for patients with severe health conditions^28^. Therefore, pharmacogenetic data and insights from our study are relevant for public health in Mexico and for Mexican-American individuals in the United States, as current standard opioid dosing guidelines may inadvertently increase the risk of adverse effects in these populations. Our findings point to a potential benefit of tailoring opioid prescriptions based on genetic profiles, particularly in individuals with origins from southern states such as Chiapas, Oaxaca and Yucatán. Further clinical studies are needed to evaluate the impact of pharmacogenetic testing on patient outcomes.

In these types of decision-making scenarios, while continental ancestry proportions can provide some guidance, they cannot alone predict the genetic risk of admixed individuals in populations with complex demographic histories. To capture the finer scales of this complexity, we demonstrate that a local ancestry analysis approach is needed so that the relationship between allele frequencies and the different ancestral components can be disentangled. By applying this approach, we were able to calculate asF nationwide for 42,769 biomedically relevant variants, revealing patterns that variation in genome-wide ancestry proportions cannot explain. This resulted in the detection of substantial local differentiation patterns, highlighting how subcontinental structure, particularly within the Indigenous American ancestry component, has a crucial role in shaping the genetic diversity of clinically relevant variants.

A major case of a differentially distributed clinical variant under an ancestry-specific lens is the SNP rs4149056 in the *SLCO1B1* gene, which is strongly linked to statin metabolism in Mexican populations, with C allele carriers being intermediate or slow metabolizers of atorvastatin^29,30^. Statins are widely prescribed to reduce LDL cholesterol and prevent cardiovascular disease and are being used by an estimated 7.9 million people in Mexico^31^. While effective and affordable, their metabolism is influenced by genetic variation, reflected in substantial allele frequency differences across continental and subcontinental groups^32^. By showing the remarkably higher values of Indigenous asF of this allele, particularly in the Yucatan and broader Mayan region, we demonstrate that the source of this geographic difference was only resolvable through local ancestry inference, which can help recover hidden substructure patterns in admixed populations. Indeed, when looking at this allele in source populations from an independent cohort of self-identified Indigenous individuals in Mexico^10^, we confirmed a significant differentiation in the southeast (Fig. 2c and Supplementary Fig. 9). Given the high prevalence of the C allele in the Mayan region, and the high burden of coronary heart disease as a leading cause of death in Mexico, national guidelines should consider adjusting statin dosing in these populations based on genetic testing or alternative statins (for example, pravastatin and fluvastatin) should be prioritized to reduce the risk of myopathy and rhabdomyolysis associated with long-term exposure.

On the other hand, our ancestry-specific analyses also revealed that many high-evidence pharmacogenetic variants are rare or absent in Indigenous ancestry segments, highlighting the limits of applying European-based guidelines to diverse populations like Mexico. As reported in other Indigenous American and Latin American cohorts^23^, the apparent absence of known variants might reflect the presence of yet-undiscovered rare alleles associated with poor or ultrarapid drug metabolism—variants that remain uncharacterized in current PGx panels. This highlights the need to diversify PGx discovery efforts, as clinically actionable variants may differ markedly—or be entirely absent—in underrepresented populations, particularly those with substantial Indigenous or African genetic contributions.

In addition to PGx variants, we analyzed clinically actionable variants from the ACMG list, which are critical for early diagnosis, prevention and management of genetic diseases. These variants provide important opportunities for precision public health, as their detection can directly inform newborn screening and targeted interventions. For example, the pathogenic allele identified in the *BTD* gene associated with biotinidase deficiency was found exclusively within Indigenous ancestry segments and showed strong regional variation. Indeed, its occurrence in Indigenous ancestry segments is centered exclusively on the Yucatan, suggesting a source in the Mayans, native to that region. Its frequency in the MXB, stemming largely from the Yucatan, was approximately 0.25%, while in a separate cohort with available genomic data for Mexican individuals, the Mexico City Prospective Study (MCPS)^33^—a predominantly urban cohort restricted to Mexico City—it had a sixfold lower frequency (~0.04%), also exclusively within Indigenous segments. Given that biotinidase deficiency is treatable via early biotin supplementation and is included in Mexico’s national newborn screening program^34^, identifying subpopulations with higher carrier frequencies could support the development of targeted screening strategies to prevent irreversible neurological damage.

The MXB was instrumental in uncovering these fine-scale patterns, demonstrating the importance of such resources in advancing genetic research and precision health for highly diverse and yet understudied populations. The local ancestry analytical approach allows for the identification of variants with higher frequencies in Indigenous segments and with geographic variation across different Indigenous sources, suggesting particular Indigenous groups and regions with a greater prevalence that might otherwise remain undertested. Such insights can help pinpoint target populations for follow-up studies. By compiling large-scale, diverse genetic datasets, biobanks like this help researchers to explore the genetic basis of complex diseases, uncover new therapeutic targets and enhance the accuracy of personalized medicine^35^. Our analysis of biomedically relevant variants in the MXB underscores the complexity of genetic variation across Mexico, necessitating a case-by-case approach to fully understand these patterns. To address this diversity, we developed MexVar, a user-friendly platform that enables the real-time exploration of allele frequencies by integrating genome-wide and ancestry-specific data. This tool can help address the challenges of analyzing complex admixed genomes, removing the dependency on extensive bioinformatics infrastructure and making genetics research more accessible to nongenomics researchers and clinicians, especially in resource-limited settings.

While the MXB offers the broadest geographic coverage of any genomic resource in Mexico, it does not fully capture the genetic diversity of the entire population, and some groups—particularly smaller or more geographically restricted Indigenous communities—may be underrepresented. We acknowledge this limitation and have addressed it to the best of our ability with the existing data. Expanding biobanking and whole-genome sequencing efforts will enable the discovery of rare and population-specific variants that are currently not captured by the MEGA array, which is an affordable assay that includes clinically relevant variants.

MexVar aims to make these data accessible locally and globally, empowering precision health research in Latin America, where genetic histories are often distinct from the global average. Enhancing our understanding of the frequencies of PGx alleles and clinically actionable variants across a broader spectrum of biogeographic origins will strengthen testing panels and improve treatment outcomes in diverse populations^36,37^, including, but not limited to, underrepresented ancestries in the Global South. Clinical testing recommendations based solely on the Hispanic/Latino label are insufficient for personalized medicine, as they neglect to consider this substantial regional, national and local genetic variation across Latin America. As we continue to push the boundaries of precision medicine, initiatives like the MXB and implementations such as MexVar will have a crucial role in delivering personalized and equitable healthcare solutions, ensuring that the benefits of genomics research extend to all populations.

## Methods

### Human participants and ethics

This work did not involve new recruitment of human research participants. Demographics of previously recruited human participants included in the MXB project are described in ref. ^11^. Ethical approval was obtained from the Institutional Review Board of the National Institute of Public Health (INSP; approvals CI1479 and CB1470) for the genetic characterization of samples from the 2000 National Health Survey (ENSA 2000). Participants were enrolled through informed consent and extensive community engagement nationwide. National Health Surveys in Mexico have been conducted periodically since 1988, so the population is highly participative and receptive to household visits by INSP staff and fieldwork teams. Sampling and biobank maintenance were carried out by INSP, while genomic data were generated at the Cinvestav Research Center in Mexico. The data have been analyzed jointly, fostering interinstitutional collaboration and local leadership among Mexican researchers and trainees.

### MXB dataset

To explore and catalog biomedically relevant variants within Mexican populations, we used the genetic information generated by the MXB Project^11^. It currently includes data for 6,011 individuals from all 32 states across Mexico recruited as part of the 2000 National Health Survey (ENSA 2000), and genotyped at 1.8 million variants on the MEGA^39^. The MEGA array provides extensive coverage of clinically relevant variants associated with disease and PGxs, ensuring the representation of diverse populations. Its design incorporates over 500,000 variants linked to clinical research, drawing from major variant annotation databases, including ClinVar, OMIM, the GWAS Catalog, PharmGKB, ACMG, the Clinical Pharmacogenetics Implementation Consortium and gnomAD. This comprehensive integration offers a robust framework for identifying variants with established or potential clinical significance.

Participants in the ENSA 2000 were selected using a probabilistic, multistage, stratified and clustered sampling design to ensure national representativity across 32 states of Mexico. The survey targeted civilian, noninstitutionalized individuals and collected household, health and sociodemographic data through structured interviews conducted by trained personnel. Biological samples, including serum and buffy coats, were obtained from 43,085 individuals aged 20 years or older. This cohort has been used in various epidemiological and genetic studies, offering a comprehensive resource for assessing health determinants at both the individual and population levels. The inclusion of individuals from rural and remote areas further strengthens the dataset’s utility in investigating genetic diversity and its implications for health disparities in Mexico. For more details, see ref. ^40^.

Samples for the MXB project were selected to maximize both geographic coverage and representation of Indigenous ancestries. The 6,011 genotyped samples are distributed across 898 recruitment sites throughout Mexico, ensuring an average sample size of five to ten individuals per locality, regardless of population density. Each state has, on average, 188 individuals, ranging from 86 to 309. The number of individuals per state is shown in Extended Data Fig. 1. The selection process prioritized individuals who reported speaking an Indigenous language (1,055), followed by random selection until budget limitations were reached. For further details, see ref. ^11^.

### Curation of biomedically relevant variants

We focused on known variants within our dataset that are relevant to human health. To identify these variants, we used data from the following four main databases: ClinVar, GWAS Catalog, PharmGKB and OMIM^12–15^. The complete datasets were downloaded directly from their respective websites in March 2023. We parsed these databases to extract biomedically relevant variants, including their variant identifiers, chromosomal locations, genetic positions, effect directions, levels of evidence, clinical significance, drug associations, associated phenotypes and related genes. We then intersected these variants with the 1.8 million variants directly genotyped for the MXB cohort. For a subset of the analysis, and for the MexVar app, variants with a MAF of less than 5% were filtered out using PLINK^41^ with the MAF option set to 0.05, ensuring that our analysis focused on variants exhibiting meaningful frequency patterns within the Mexican population. After merging and filtering, we retained 42,769 variants. A detailed summary of these selected variants is provided in Supplementary Table 1.

### Ancestry and population descriptors

‘Genetic ancestry’, as used in this study, is a statistical construct based on the genetic similarity that an individual shares with a given reference panel of source populations, reflecting their potential ancestors. In contrast, ‘race’ and ‘ethnicity’ are social constructs used to group people based on perceived physical, geographical, cultural or other social characteristics. In the analyses presented here, we exclusively refer to genetic ancestry as described above, except when mentioned otherwise. Notably, an individual’s assigned genetic ancestry is not equivalent to, and does not invalidate, how that individual self-identifies.

In this study, we distinguish between two levels of genetic ancestry: continental and subcontinental.

Continental ancestry refers to broad ancestral groupings based on large-scale worldwide population structure; this study includes Indigenous American, African and European components. These proportions were inferred using global reference panels and represent the primary axes of genetic variation relevant to populations across Mexico.

Subcontinental ancestry, on the other hand, captures the finer-scale structure within the continental component. While continental ancestry reflects large-scale population groupings, subcontinental ancestry refers to the regional genetic differentiation that arises from long-term demographic, cultural and geographic isolation within continental landmasses. For example, within a continental ancestry, such as Indigenous American, there exists substantial genetic heterogeneity between regional populations due to historical separation, founder effects and limited gene flow. Accounting for this substructure is essential for accurately characterizing patterns of genetic variation, since different subcontinental contributions can lead to significant differences in risk allele frequencies with medical implications, even among individuals with similar continental ancestry proportions.

### GLMs

We used linear models to investigate the influence of genetic background (Indigenous American and African ancestry) and geographic factors on the variation observed in biomedically relevant variants. Before modeling, these variables were standardized in R to reduce scale-related biases. Subsequently, GLMs were used in R^42^ to analyze the associations between the standardized predictor variables and genetic variations. Coefficients and *P* values were calculated to determine the statistical significance of each predictor variable.

### Local ancestry analysis

#### Local ancestry inference

To further investigate the influence of ancestry on the incidence of biomedically relevant variants in individuals within the MXB cohort, we used the Gnomix software from ref. ^43^ to infer local ancestry tracts. We used a *k* = 4 model, which assumes the presence of four distinct genetic groups. Reference populations were selected to represent the major continental genetic ancestries in Mexico^11^—African (Afr), European (Eur), East Asian (Eas) and Indigenous American (Ind; Supplementary Fig. 14). This approach allowed us to accurately characterize the genetic contributions from these ancestries within the cohort.

Reference populations were taken from the Human Genome Diversity Project^44^, the Population Architecture using Genomics and Epidemiology study^45^, and individuals from the MXB. We used the same number of individuals in each reference population to mitigate potential bias towards a particular ancestry. Sixty individuals were randomly chosen for each of the four populations. For the African component, we selected individuals self-identifying as Bantu, Mandenka and Yoruba; for the European, we selected populations from Western Europe, individuals self-identifying as French, Italian and Orcadians; and for East Asian, we used a combination of individuals identifying as Han and Japanese. For the Indigenous people from the Americas, we integrated genetic information from various groups, including individuals self-identifying as Mixe, Surui, Puno, Zenu and Indigenous populations in Honduras. We also included individuals from the MXB who exhibited more than 98% Indigenous ancestry based on unsupervised ADMIXTURE analysis^46^.

We used PLINK^41^ to merge the datasets and retained only the intersecting biallelic variants, excluding triallelic variants and those with genotype missingness <5%. We ran an ADMIXTURE analysis to corroborate the homogeneity of our reference panel (Supplementary Fig. 14). We ran Gnomix with the default parameters, with the exception of setting ‘inference to best’ and ‘phase’ to FALSE.

#### Local ancestry accuracy

We used Gnomix to estimate local ancestry tracks due to its higher accuracy compared to other programs like RFmix, as stated in the main Gnomix paper^43^. This paper also evaluates accuracy over time and reports that for ancestries traced back up to 20 generations, Gnomix maintains an accuracy of over 93% on array data. Given that our dataset primarily captures ancestry within the past 16 generations (assuming an average of 30 years per generation and admixture starting approximately 500 years ago), Gnomix is well-suited for our analysis.

We also conducted a simulation to evaluate the impact of local ancestry inference errors on allele frequency estimation. We simulated chromosomes with ancestry proportions reflecting those observed in the Mexican population (Afr = 4, Ind = 65, Eur = 30, Eas = 1). In this simulation, alleles were assigned frequencies specific to each ancestral background (for example, Ind = 0.1). To model errors in local ancestry prediction, we incorporated the confusion matrix derived from the Gnomix results. Using the predicted ancestry, we then estimated asF across a range of MAFs, using a sample size comparable to that of the MXB. Overall, in our simulations, we found that local ancestry inference errors had minimal impact on the estimation of asF (Supplementary Fig. 15). When training the model with our own data, we achieved a mean estimated accuracy of 94.97% across all chromosomes.

#### Estimation of asF

To estimate asF, we used a customized pipeline approach (Fig. 5) that involved creating a masked Variant Call Format (VCF) file. This approach uses predicted local ancestry inferences, in which variants that do not match the specified ancestry are designated missing. Subsequently, allele frequencies are computed from these ancestry-masked VCF files using VCFtools with the --freq option^47^. asF are then calculated as the ratio of alleles corresponding to a given ancestry over the total number of alleles for that ancestry, as detailed in the following formula:

$${\mathrm{asF}}_{x}=\frac{p\,\mathrm{alleles}\,\mathrm{in}\,x\,\mathrm{ancestry}}{\mathrm{total}\,\left(p+q\right)\,\mathrm{alleles}\,\mathrm{in}\,x\,\mathrm{ancestry}}$$

where *x* is the given ancestry.

#### asFst

To accurately quantify genetic differentiation between the states in Mexico, we calculated asFst using a customized ancestry-specific mask to filter variants for each specific ancestry. We provided a VCF file with missing alleles from other ancestries to VCFTOOLS^47^ using the --weir-fst-pop argument, performing this analysis separately for European and Indigenous local ancestry masks. This approach allowed us to quantify genetic differentiation between these populations accurately. asFSt values were calculated for all biomedically relevant variants with MAF > 0.05

### Clinically relevant and actionable variants

To identify variants with established clinical relevance, we defined a subset referred to as the clinically relevant and actionable variants. This subset was curated to include only variants with strong evidence for medical actionability based on current clinical guidelines and expert consensus. The following criteria were applied:

1. PGx variants—we included variants annotated in the PharmGKB database at level 1A, 1B, 2A and 2B corresponding to gene–drug associations supported by clinical practice guidelines
2. Pathogenic variants in medically actionable genes—we identified all pathogenic and likely pathogenic variants without conflicting interpretations reported in the ClinVar database (accessed 30 April 2023) occurring in genes listed in the ACMG SF (v3.2; ref. ^48^). The guideline list includes 81 genes deemed to be medically actionable, of which 28 are associated with hereditary cancer, and the remaining 53 genes are associated with cardiovascular, metabolic and other genetic conditions for which there are available medical interventions that can prevent or reduce morbidity and mortality due to these conditions.

Notably, no allele frequency threshold was applied to this subset, as many clinically relevant variants are rare but nonetheless important for diagnosis or therapeutic decisions.

This curated subset was used to illustrate the ancestry-specific distribution of clinically actionable variants within the MXB cohort. Summary statistics—including the number of variants, genes and distribution across ancestries—are provided in Fig. 1 and Extended Data Fig. 3.

### PharmGKB annotations

We obtained variant annotations from the PharmGKB, where each variant can be associated with multiple annotations. These annotations are categorized into six levels of evidence (4, 3, 2B, 2A, 1B and 1A; Supplementary Fig. 16), reflecting the strength of the evidence supporting the association with a particular drug response, with level 1A representing the highest evidence and level 4 the lowest. When a variant was associated with multiple drugs at different levels of evidence, we considered the drug with the highest level of evidence. Specifically, we focused on annotations with a level of evidence starting from 2B, which denotes variant-drug combinations supported by a moderate level of evidence and requires support from at least two independent publications.

For SNP rs4149056, we analyzed the top five drugs associated with it. The data for this analysis were retrieved from the PharmGKB database on 11 September 2023. For each drug, we also determined the number of studies supporting the association, providing insights into the robustness of the identified relationships (more details are available at https://www.pharmgkb.org/variant/PA166154579/variantAnnotation).

### Integration of allele frequencies of the MXB on the Shiny app

In this paper, we introduce MexVar, a robust, user-friendly web application leveraging the Shiny framework in R^49^. This graphical user interface serves as a dynamic platform for querying allele frequencies nationwide in Mexico. Our analysis relied on a comprehensive dataset from the MXB, which encompasses allele frequency distributions of biomedically relevant variants across all 32 states of Mexico and comprises 42,769 variants. Detailed information regarding this dataset is provided in Supplementary Table 1. Maps displayed here and in the MexVar app are generated using mxmaps^38^.

The application allows users to view both genome-wide and asF, thus providing an in-depth understanding of each variant’s impact relative to the ancestry. In the ancestry selection module, users can choose from five options—‘all’ (for nonancestry-specific data) and four ancestry-specific categories (European, Indigenous, African and East Asian). Detailed methodology for ancestry-specific analysis is mentioned in ‘Local ancestry analysis’.

Two mandatory inputs for the MexVar application are the rsID of a given SNP and the desired genetic ancestry used to calculate allele frequencies. Additionally, users can customize the application’s esthetic elements, such as color schemes and titles, to suit their preferences. The application processes these inputs in real time to display an output that includes a dynamic map of Mexico, where each state is color-coded according to the selected SNP allele frequency. Moreover, users can browse an additional tab within the application to explore detailed information about the SNP, including its presence in the app, the originating database, associated phenotype, gene, risk allele (if mentioned in the database) and relevant publications. Users can also download the frequency table for offline analysis, facilitating deeper investigation and data exploration.

The design is adaptable and scalable, allowing for the integration of additional data as it becomes available, such as whole-genome sequencing. This inherent flexibility facilitates ongoing improvements and the expansion of analytical scope in line with evolving datasets.

### Reporting summary

Further information on research design is available in the Nature Portfolio Reporting Summary linked to this article.

## Online content

Any methods, additional references, Nature Portfolio reporting summaries, source data, extended data, supplementary information, acknowledgements, peer review information; details of author contributions and competing interests; and statements of data and code availability are available at 10.1038/s41591-025-04100-z.

## Supplementary information

Supplementary Information Supplementary Figs. 1–16.

Reporting Summary

Supplementary Tables Supplementary Tables 1–3.

## Data Availability

Individual level genotype data were previously generated as part of The MXB project^11^, and are available at the European Genome-phenome Archive (EGA) through a Data Access Agreement with the Data Access Committee (EGA accession number for study: EGAS00001005797; dataset: EGAD00010002361 (Mexican_Biobank_Genotypes). Additionally, MXB frequency data has been uploaded as a new track to the UCSC human genome browser, and ancestry-specific frequencies are available through MexVar at https://morenolab.shinyapps.io/mexvar/.

## Code availability

The code used in the analyses can be found in https://github.com/morenolab/MexVar_Paper_Code.
