# Supplementary material for: Clinical genetic variation across Hispanic populations in the Mexican Biobank
Source: Nat Med. 2026 Jan 21;32(2):725–35. doi: 10.1038/s41591-025-04100-z (PMC12920135; doi:10.1038/s41591-025-04100-z)
Supplement: Supplementary file 1 — Supplementary Figs. 1–16. [file 41591_2025_4100_MOESM1_ESM.pdf]

nature medicine

Supplementary information https://doi.org/10.1038/s41591-025-04100-z

Clinical genetic variation across Hispanic populations in the Mexican Biobank

In the format provided by the authors and unedited

Supplementary Figure 1..2
European and Indigenous Genetic Ancestry by State in the Mexican Biobank
Supplementary Figure 2..3
Allele Frequencies of Pharmacogenomic Variants in the Mexican Biobank
Supplementary Figure 3..4
Allele Frequency Comparison of PharmGKB SNPs in African, Indigenous
and European Ancestral Segments
Supplementary Figure 4..5
Genetic Ancestry Stratification by Genotype for SNP rs2242480 in the MXB
Supplementary Figure 5..6
Alleles per Ancestry in rs2242480
Supplementary Figure 6..7
Alleles per Ancestry in rs1801265
Supplementary Figure 7..8
Ancestry-Specific Fst Values across the Genome
Supplementary Figure 8..9
Ancestry-Specific Allele Frequencies of rs4149056 across Mexican Biobank Regions
and its Association with Statin Drugs
Supplementary Figure 9..10
rs4149056 Distribution in Native Mexican Diversity Project Panel Replicates
the one in the MXB in Indigenous Segments.
Supplementary Figure 10..11
Allele Frequency of the rs3812718
Supplementary Figure 11..12
Geographic Distribution of African Ancestry in Mexico.
Supplementary Figure 12..13
Association between Global Ancestry, Local Ancestry and Fst
Supplementary Figure 13..14
Difference in allele frequency between ancestries (Ancx,y) across chromosomes
Supplementary Figure 14..15
Admixture Plot of Continental References used for Local Ancestry Calls
Supplementary Figure 15..16
Simulation of Local Ancestry Inference Error Impact on Allele Frequency Estimation.
Supplementary Figure 16..17
Distribution of PharmGKB Variants Present in the Mexican Biobank (MXB)
by Level of Evidence.

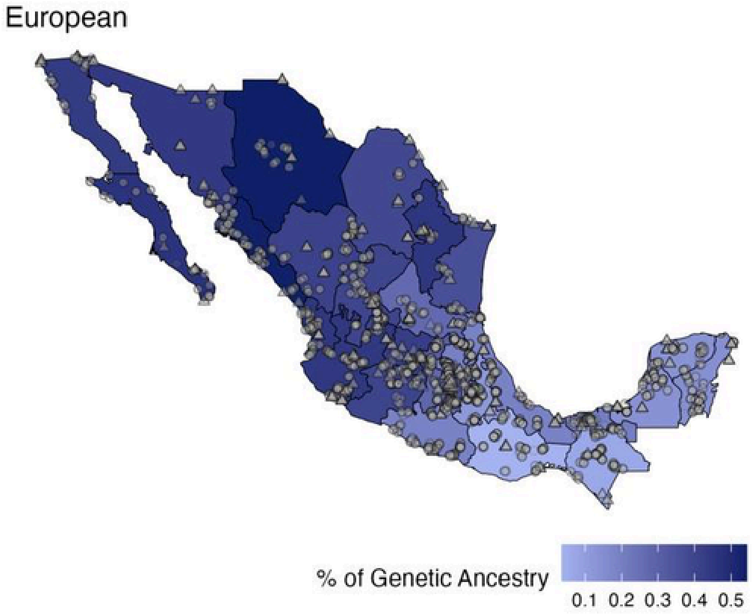

Supplementary Figure 1. European and Indigenous Genetic Ancestry by State in the Mexican Biobank
Map of Mexico showing the sampling locations of the 6,011 individuals included in the Mexican Biobank (MXB) dataset. The map highlights the 32 states of Mexico, color-coded by their mean European or Indigenous genetic ancestry. In the European component the pattern inversely mirrors that of the Indigenous component, with the northern states exhibiting higher levels of European ancestry, in contrast to the southern states, which display lower levels.

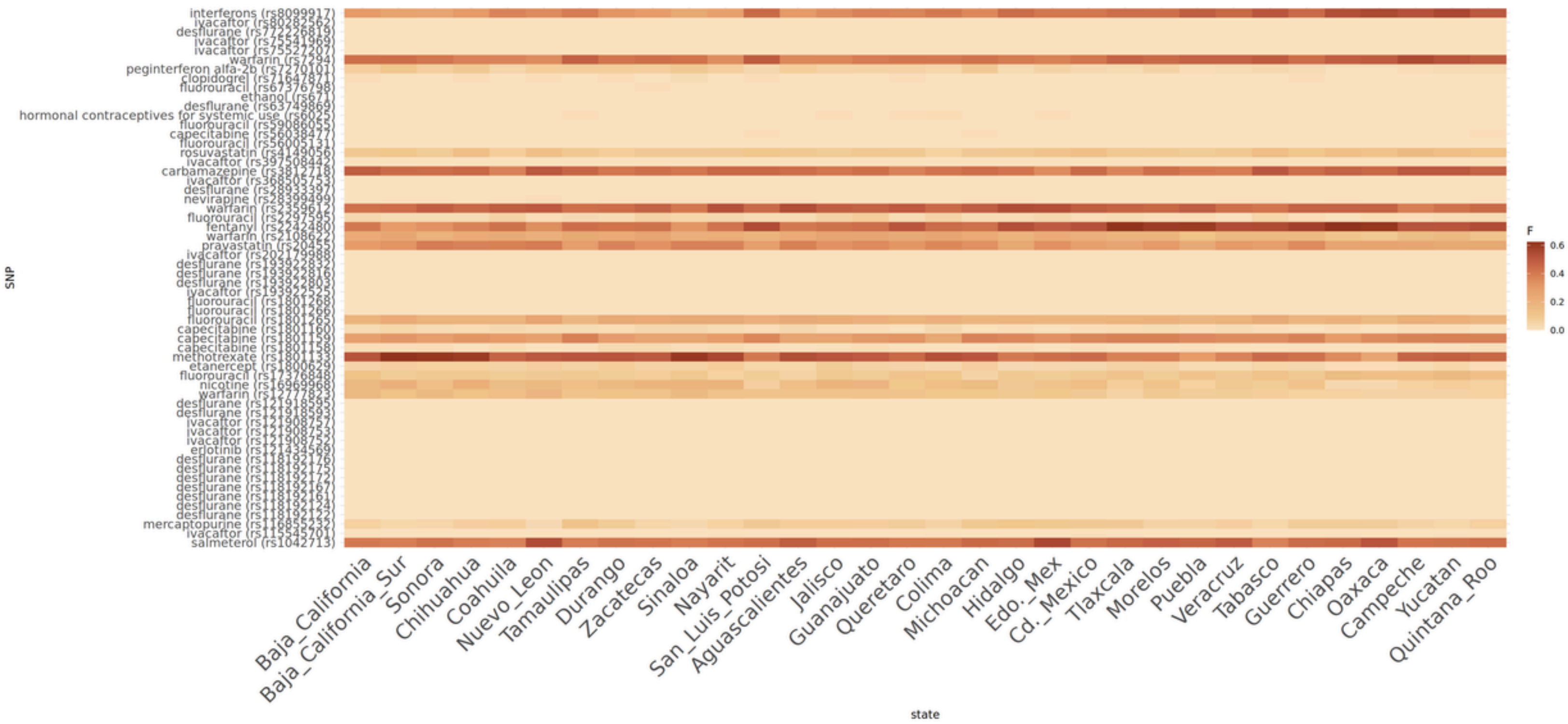

Supplementary Figure 2. Allele Frequencies of Pharmacogenomic Variants in the Mexican Biobank

Heatmap displaying the allele frequencies of clinically relevant pharmacogenomic variants, curated from the PharmGKB database, across different Mexican states. The states are sorted by geographic location (north to south-east)

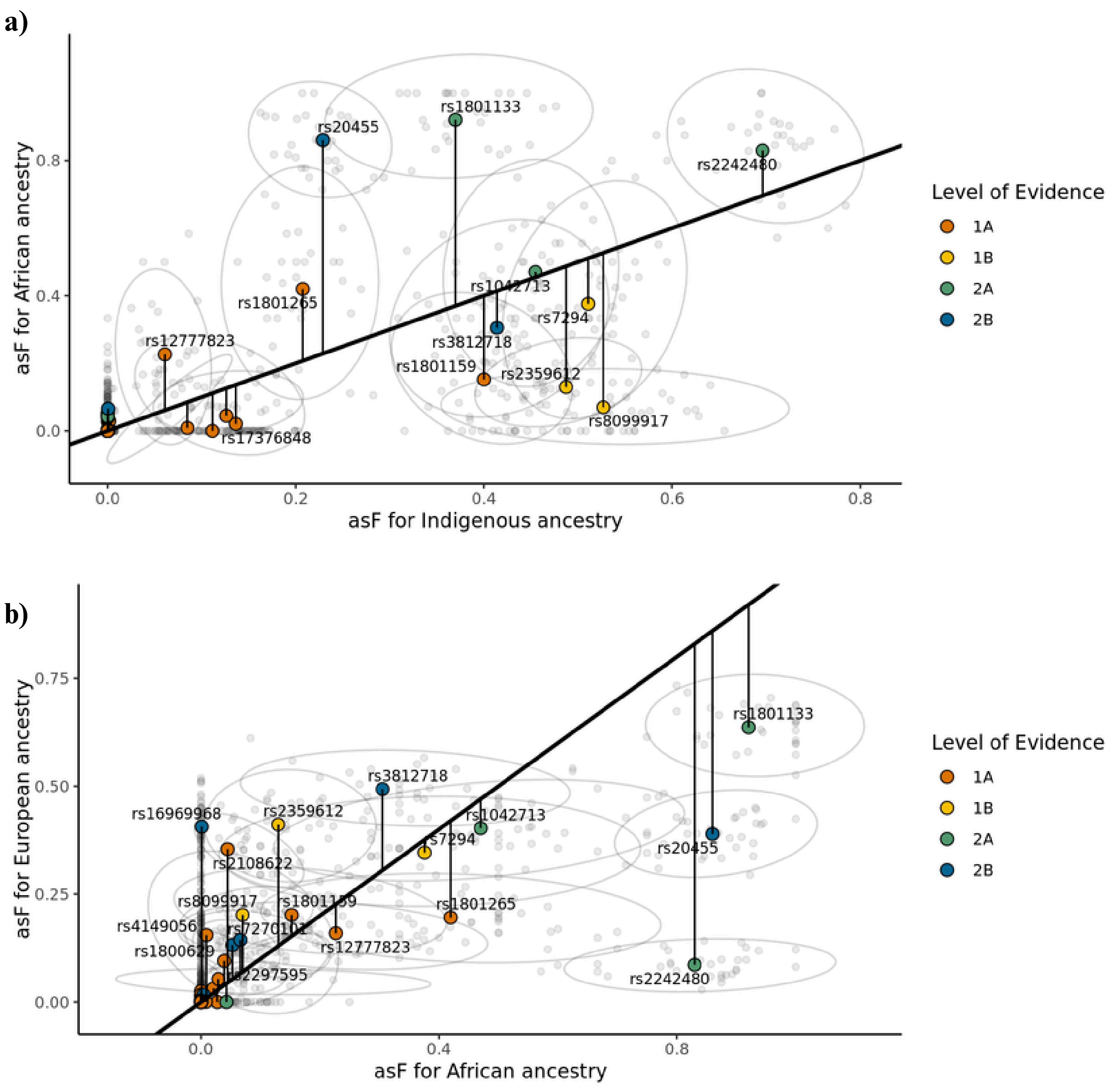

Supplementary Figure 3. Allele Frequency Comparison of PharmGKB SNPs in African, Indigenous and European Ancestral Segments

Ancestry-specific allele frequency (asF) for 58 SNPs with higher levels of evidence associated with drug responses, as documented in the PharmGKB database. asF calculate allele counts only within chromosomal segments inherited from the ancestry of interest. The gray points on the graph represent the asF for each SNP in each of the 32 states, while the colored points represent the nationwide asF. The distance of each point from the diagonal line indicates the degree of enrichment toward one ancestry. The color-coding in the graph corresponds to the level of evidence associated with each SNP. The asF data is presented for 3 distinct ancestries: a) asF African vs asF Indigenous. b) asF European vs asF African.

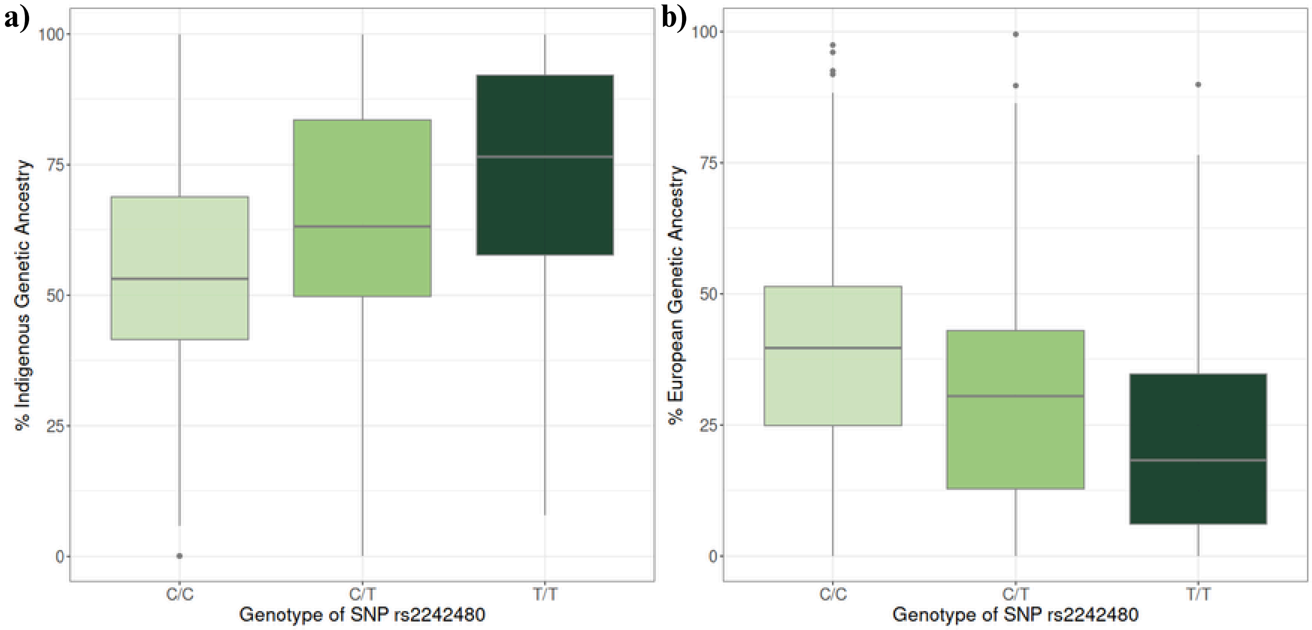

Supplementary Figure 4. Genetic Ancestry Stratification by Genotype for SNP rs2242480 in the MXB

a) Percentage of Indigenous genetic ancestry in individuals from the Mexican Biobank with different genotypes of SNP rs2242480. b) Percentage of European genetic ancestry in individuals from the Mexican Biobank with different genotypes of SNP rs2242480.

a)

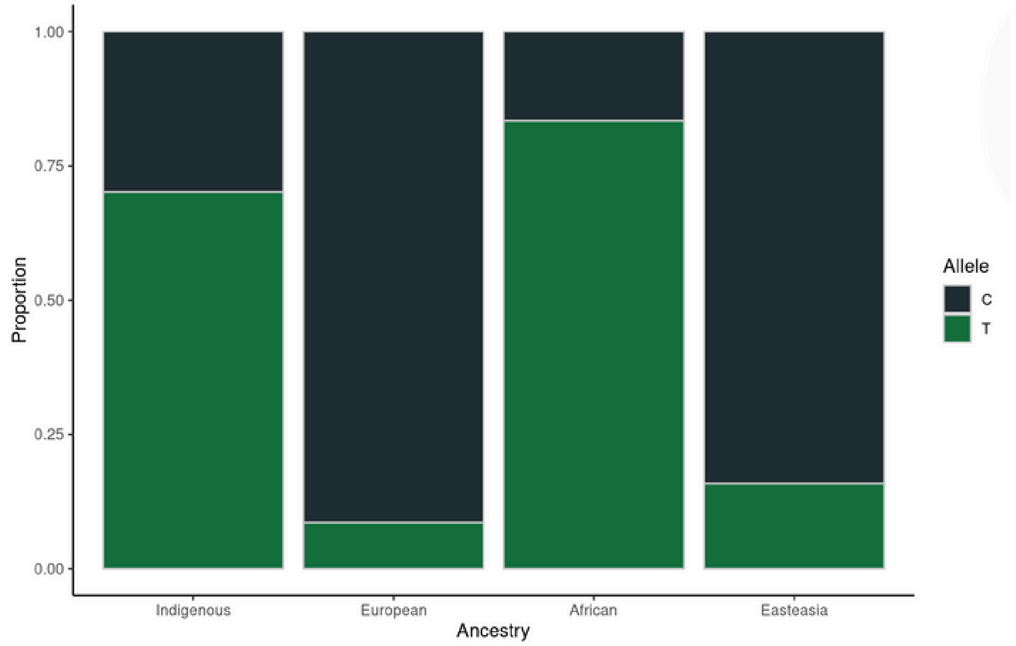

b)

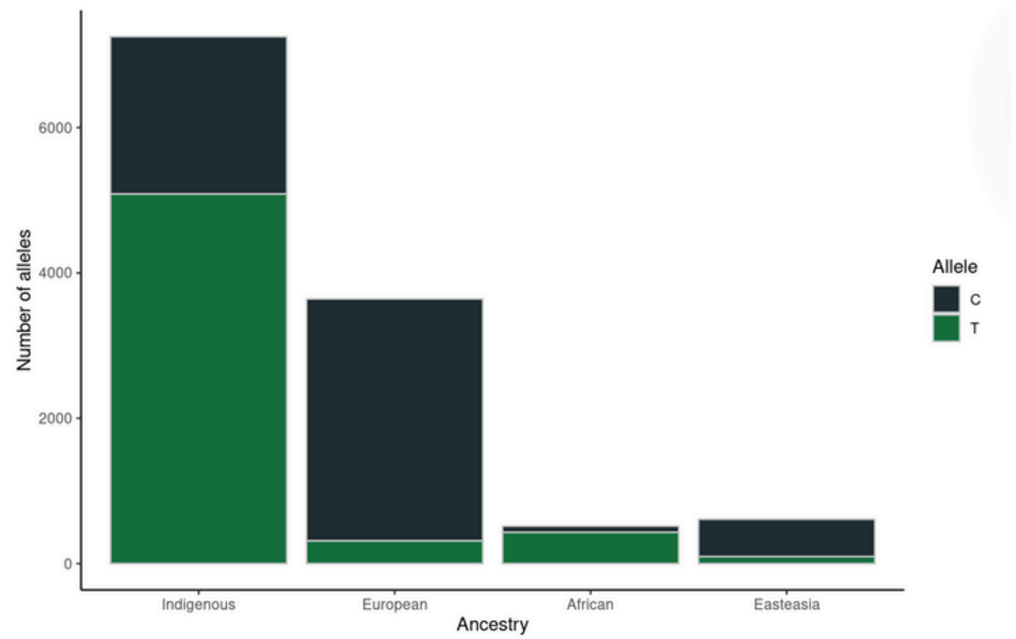

Supplementary Figure 5. Alleles per Ancestry in rs2242480

a) Proportion of each allele of rs2242480 aggregated by the ancestry to which each genomic segment is assigned. The allele T shows higher proportions in Indigenous and African ancestry backgrounds. b) Total number of alleles per ancestry group. This panel highlights the allele count distribution across ancestries.

a)

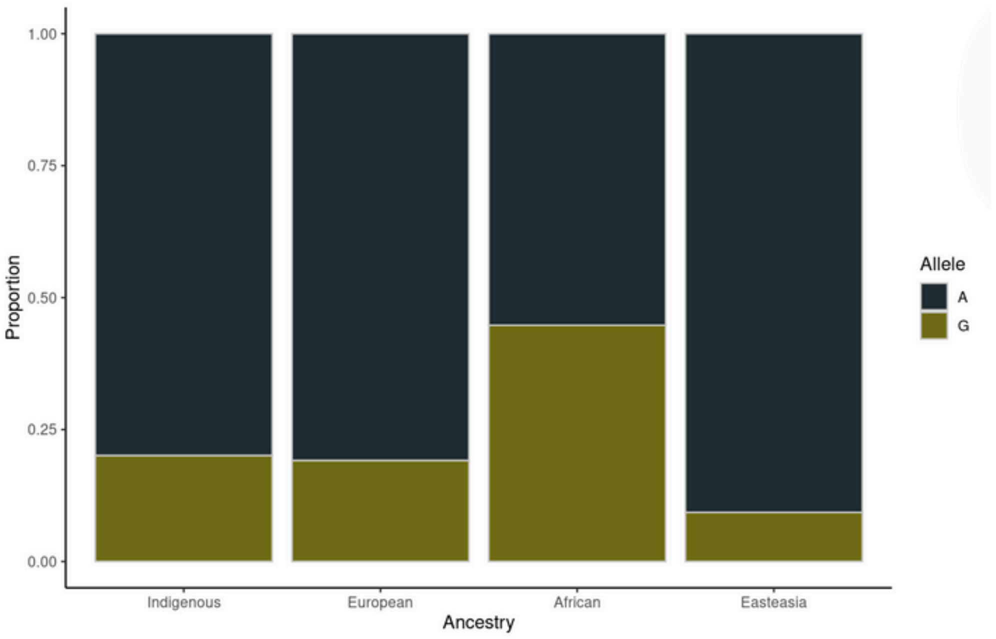

b)

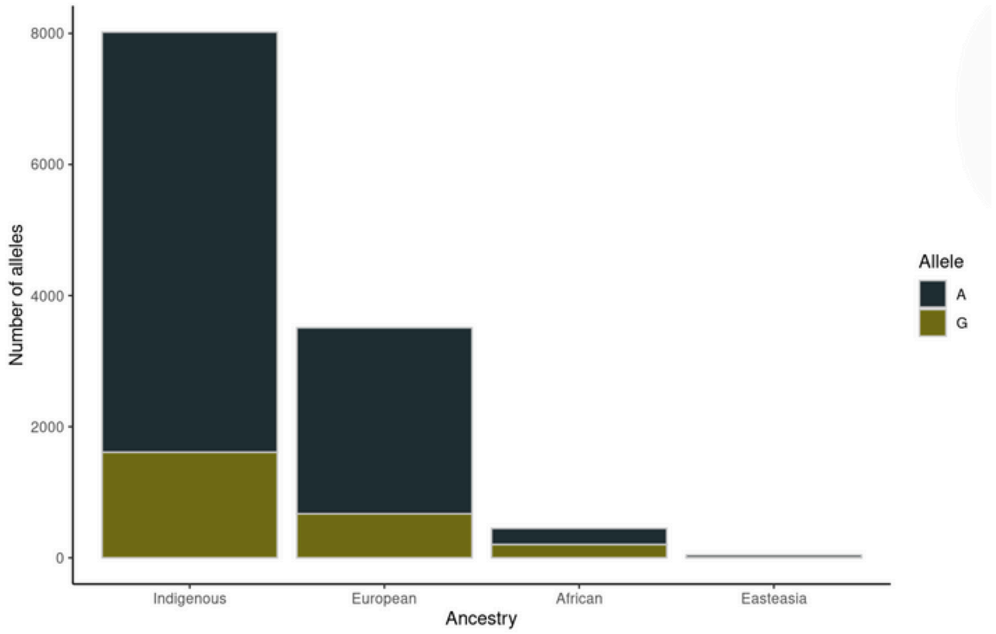

Supplementary Figure 6. Alleles per Ancestry in rs1801265
a) Proportion of each allele of rs1801265 aggregated by the ancestry to which each genomic segment is assigned. The allele G shows higher proportions in African ancestry background. b) Total number of alleles per ancestry group. This panel highlights the allele count distribution across ancestries.

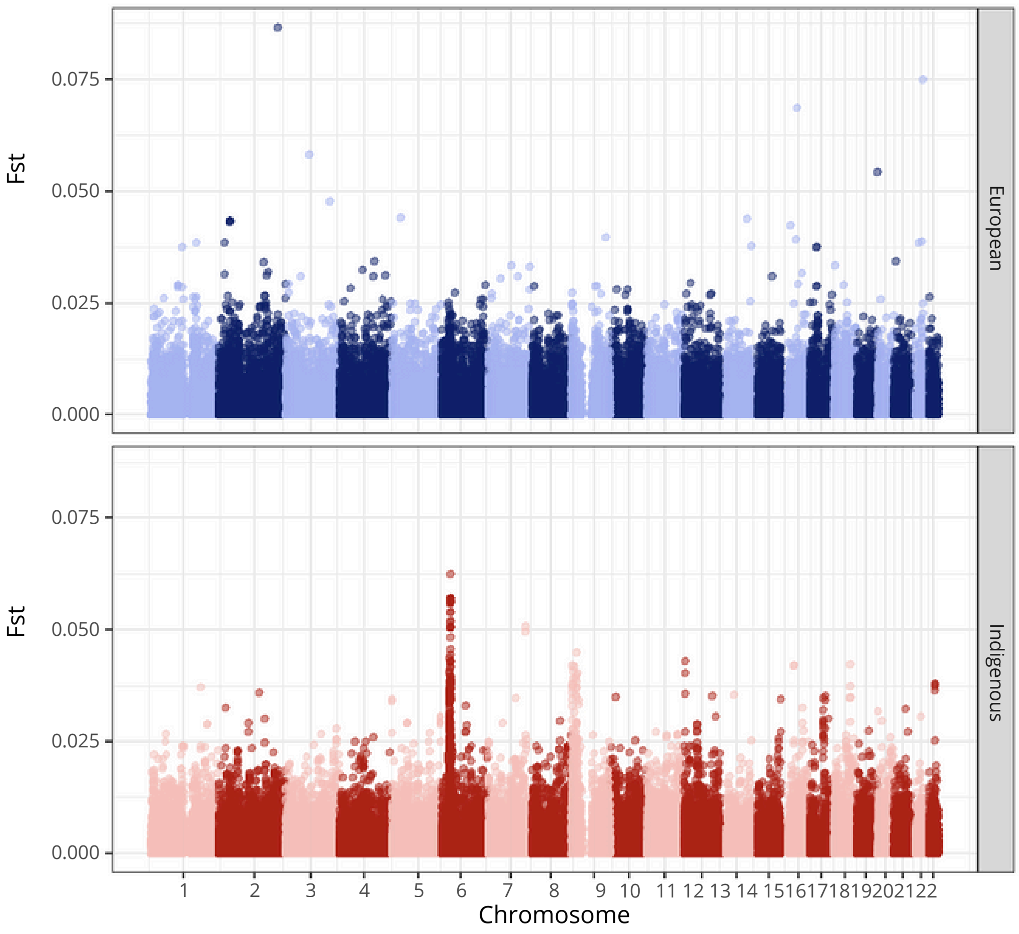

Supplementary Figure 7. Ancestry-Specific Fst Values across the Genome

Plot showing asFst across 22 chromosomes: Notably, certain regions of the genome, particularly on chromosomes 6 and 9, exhibit elevated Fst values in Indigenous segments, indicating increased genetic divergence in these regions. We found the highest density around the HLA region on Chromosome 6 driven by several SNP variants having higher asFst levels in the Indigenous component.

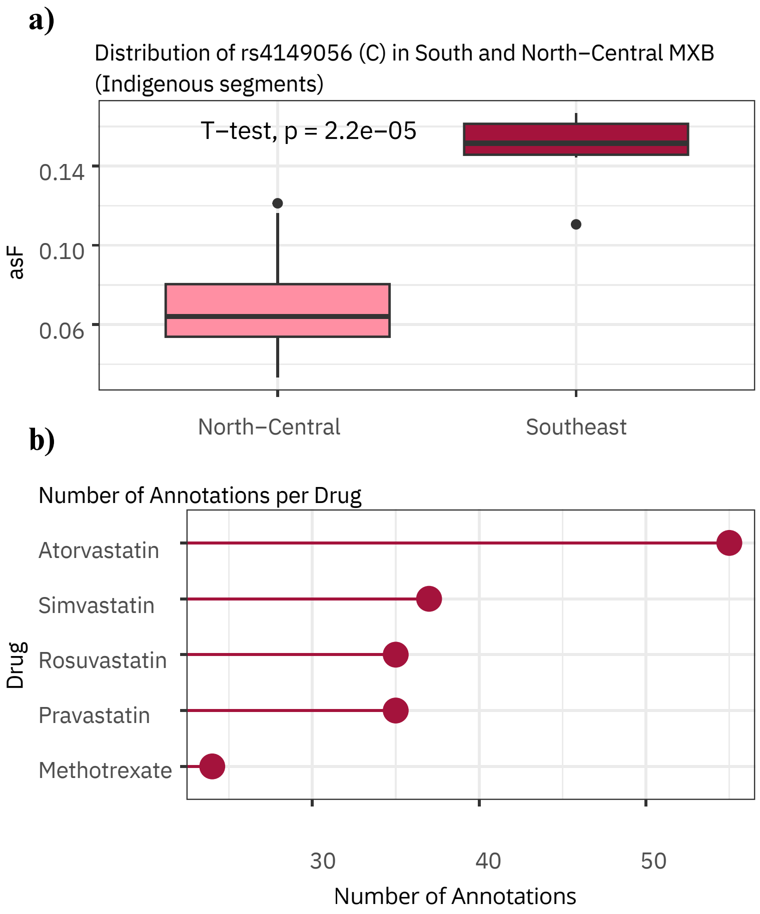

Supplementary Figure 8. Ancestry-Specific Allele Frequencies of rs4149056 across Mexican Biobank Regions and its Association with Statin Drugs

a) Box plot representing Ancestry-specific allele frequencies across the Southeast and North Central regions with significant differences (p=2.2e-05). b) Bar plot depicting the number of studies that have linked each drug to drugs associated with rs4149056 according to PharmGKB: This bar plot summarizes the number of studies that have linked the SNP with each drug.

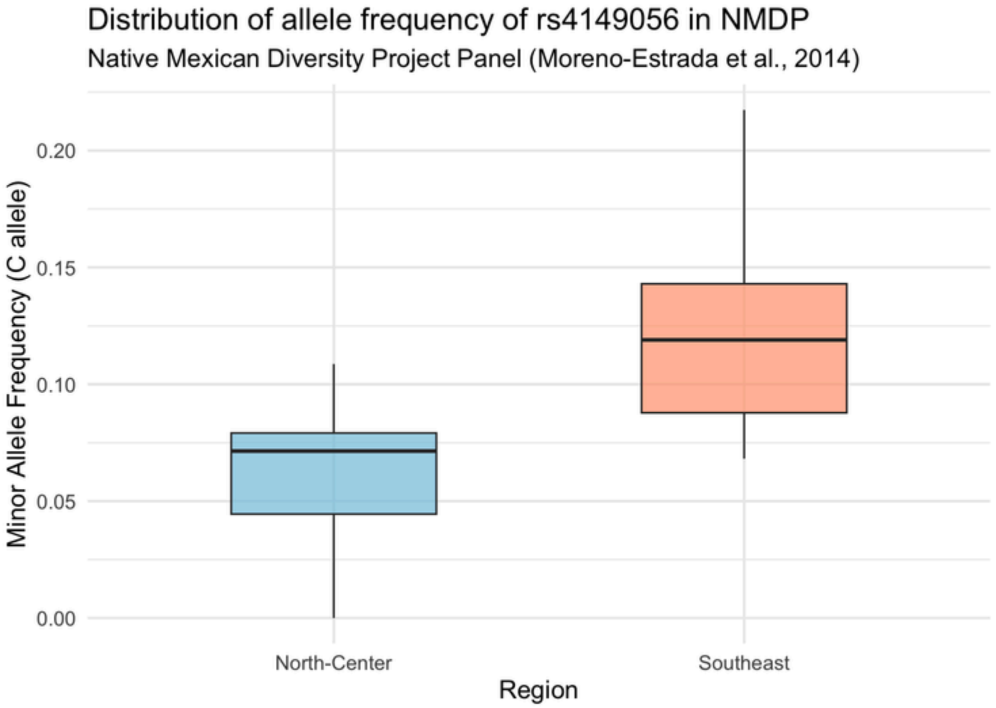

Supplementary Figure 9. rs4149056 Distribution in Native Mexican Diversity Project Panel Replicates the one in the MXB in Indigenous Segments.
Distribution of the allele frequency of the variant rs4149056 in a independent cohort the Native Mexican Diversity Project Panel (Moreno-Estrada et al., 2014) (n=454) includes Indigenous populations with self-identified ancestry, and the individuals are least admixed (>90% global indigenous ancestry). We observed a significant higher allele frequency in the southeast individuals (two-sided t-test, adjusted p = 0.035). We group each population according to the regions previously defined in (Moreno-Estrada et al., 2014), with Trique, Zapotec, Mazatec, Tzotzil, Tojolabal, Lacandon and Maya as part of the southeast region and Seri, Tarahumara, Tepehuano, Huichol, Nahuas, Purepecha and Totonac as north-center.

a)

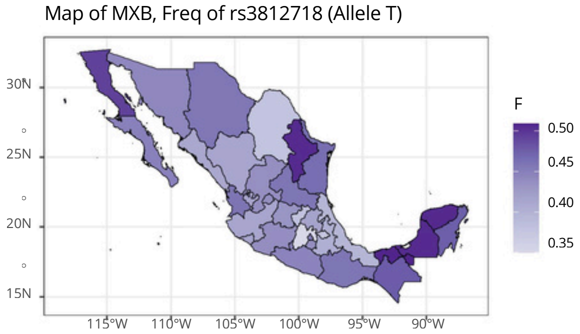

b)

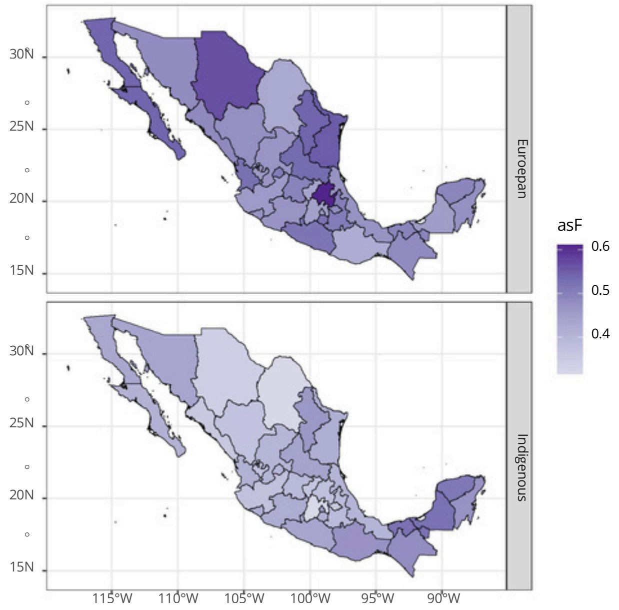

Supplementary Figure 10. Allele Frequency of the rs3812718

a) Allele frequency of the variant rs3812718 in MXB. b) Ancestry specific allele frequency for the Indigneous and European Ancestry in MXB. The pattern in a) is explain by the combination of both subcontinental variations in b).

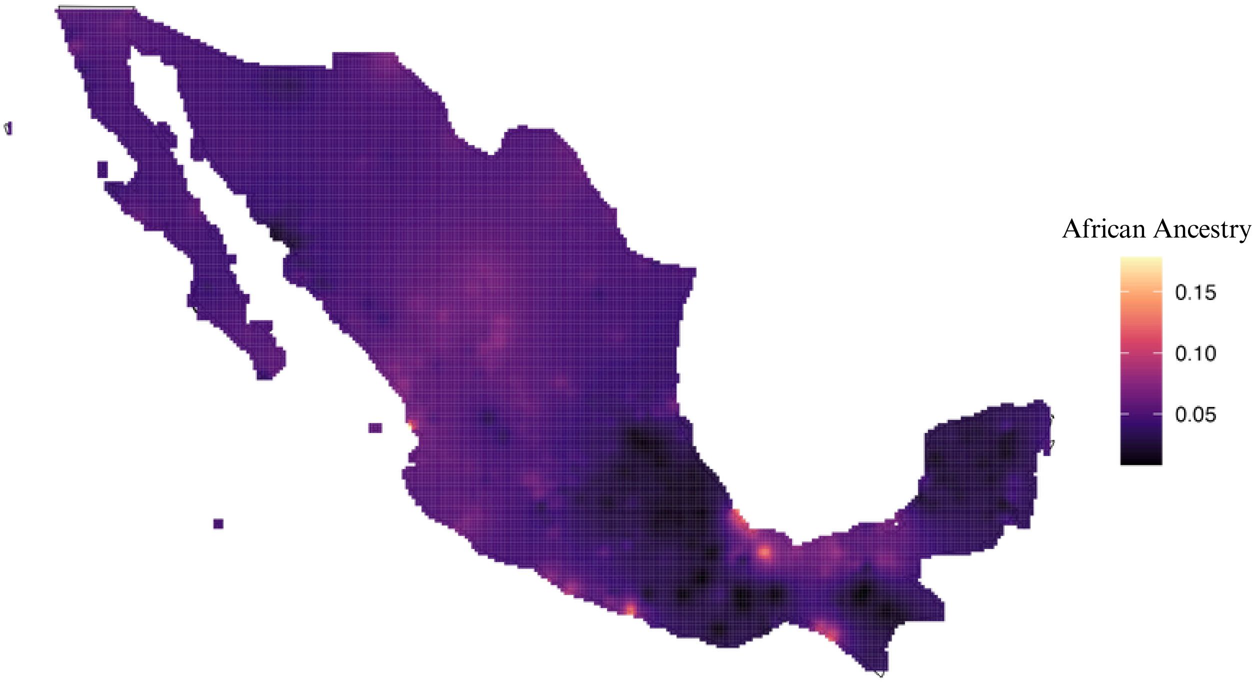

Supplementary Figure 11. Geographic Distribution of African Ancestry in Mexico
The map shows the proportion of African ancestry across Mexico, estimated using an inverse distance weighted interpolation on a smoothed grid. Smoothing was performed using data from each individual in the Mexico Biobank. Lighter colors represent higher proportions of African ancestry. Two prominent hotspots are observed along the coasts: one in the eastern region (Veracruz) and another in the western region (Guerrero), reflecting historical patterns of African admixture.

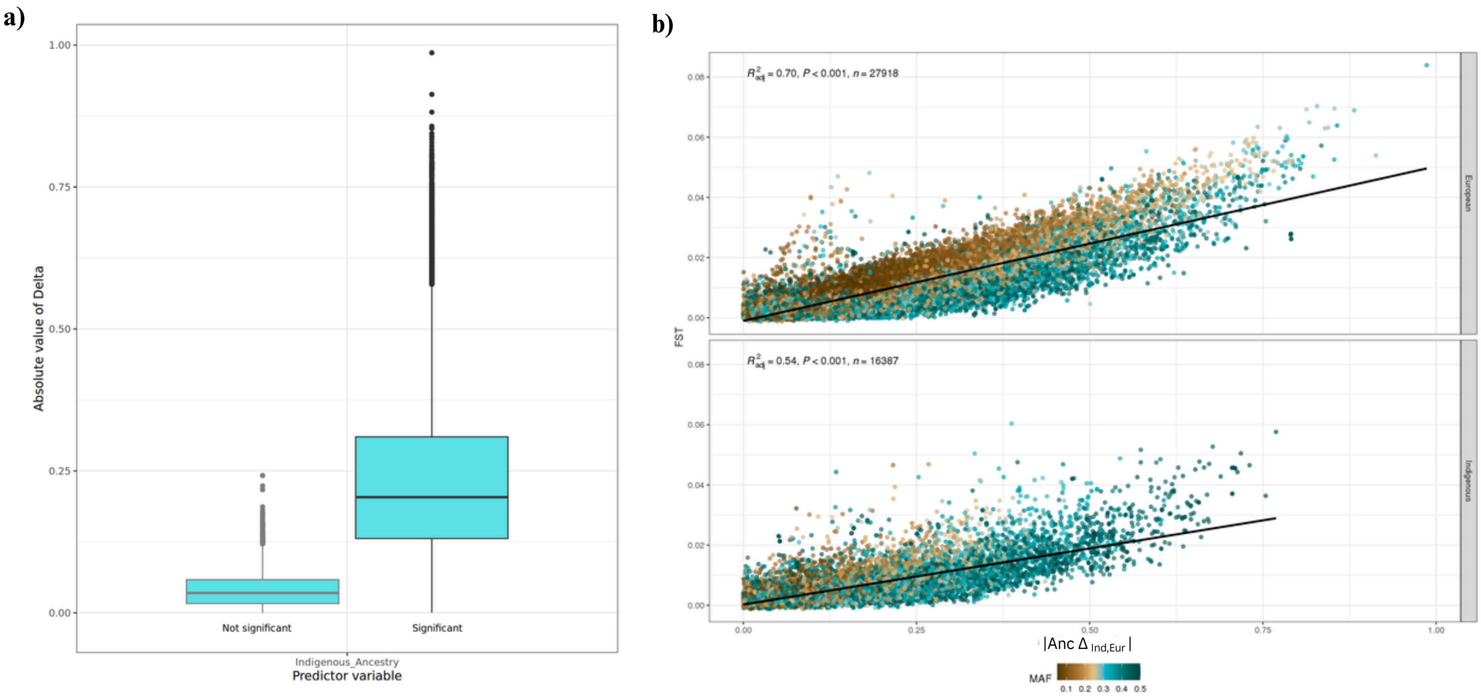

Supplementary Figure 12 Association between Global Ancestry, Local Ancestry and Fst

To assess the genetic diversity and differentiation between ancestries within our study population, we introduced a comparative measure: Difference in Allele Frequency between Ancestries (Anc x,y). This metric is defined as the difference between the ancestry-specific allele frequencies of two distinct ancestries, x and y, calculated as follows: Anc x,y = asFx - asFy where asFx and asFy represent the ancestry-specific allele frequencies for ancestries x and y, respectively. This measure enables us to quantify the degree of allelic differentiation attributed to ancestral backgrounds. An Anc x,y value close to zero indicates minimal differences in allele frequencies between the ancestries, suggesting a lower degree of genetic differentiation. Conversely, larger absolute values of Anc x,y indicates substantial differences in allele frequencies, meaning a higher degree of genetic divergence between x and y ancestries.

a) Comparison of the absolute difference in allele frequency between ancestries |Anc Δ| in variables with a significant predicted effect of Indigenous ancestry against those without. We see that ones where predicted effect of the Indigenous ancestry is significant show higher values of absolute difference in allele frequency between ancestries (t test, two sided, $t(33{,}887) = 79.1$, $p < 1\times10^{-30}$). This finding indicates that variants whose diversity is explained by global Indigenous ancestry also show a higher differential local ancestry frequency (Ind,Eur). b) Fst (as a measure of diversity across Mexican states) is correlated with |Anc Δ|. More variants were found to be enriched (higher frequency) in European ancestry. This result highlights a discovery bias due to association studies being done predominantly in European descent populations. The analysis of all biomedical variants considered in this study revealed that 63% are higher frequency in European ancestry despite the significant Indigenous ancestry within the cohort.

a)

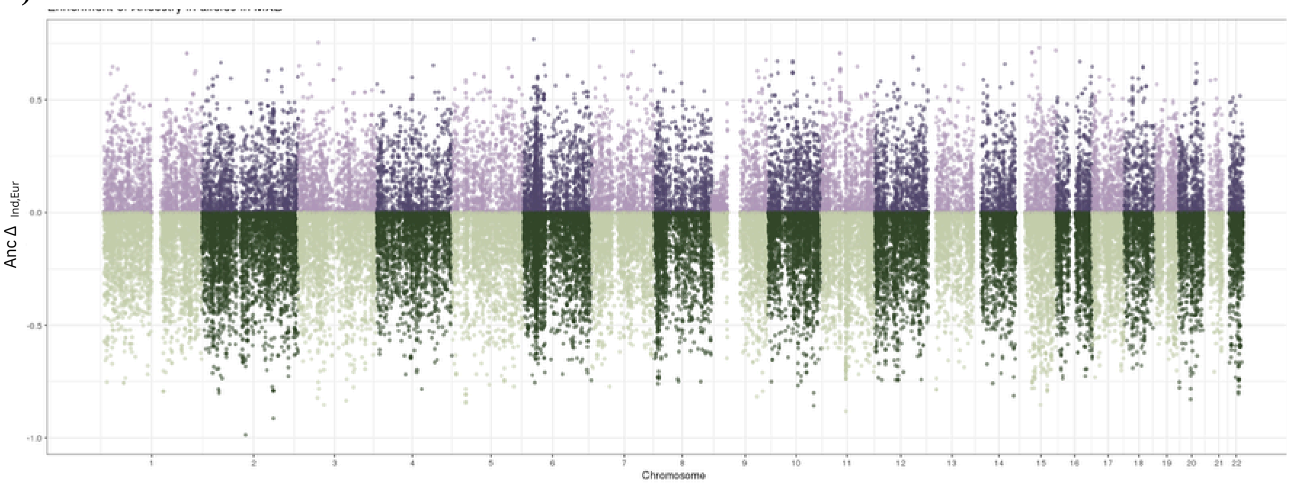

b)

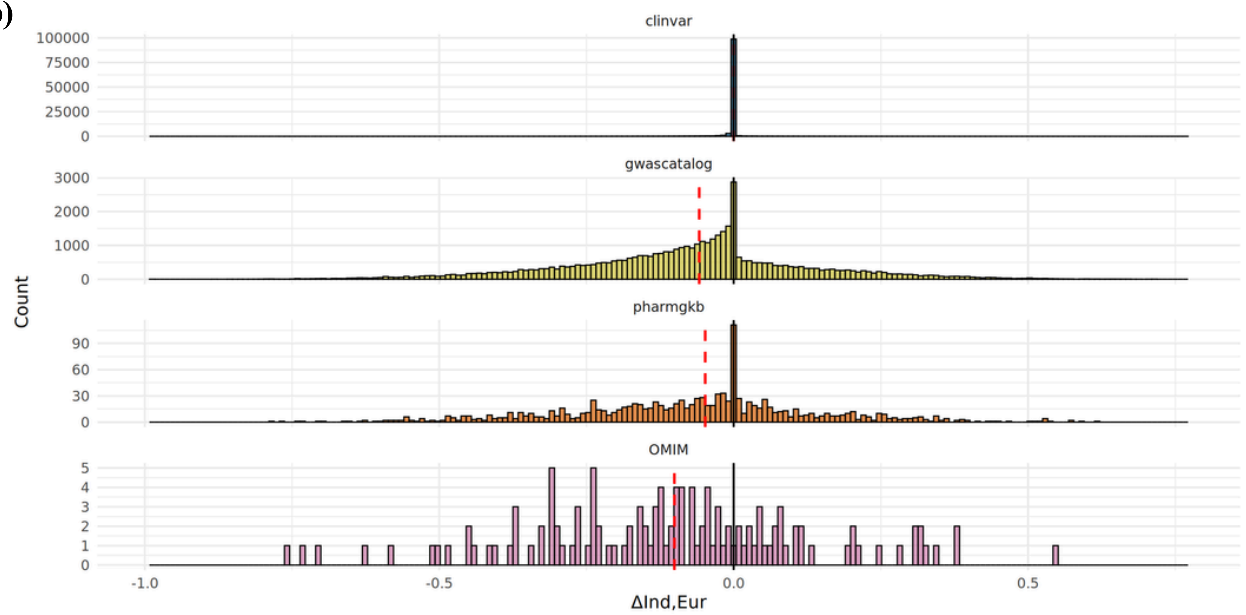

Supplementary Figure 13. Difference in allele frequency between ancestries (Anc x,y) across chromosomes

a) Variations in allele frequency across chromosomes between ancestries are depicted with the positive differences (represented in purple) indicating higher frequency in Indigenous Ancestry. On the other hand, the differences marked in green denote higher frequency in European ancestry. b) Distributions of allele frequency differences between Indigenous and European ancestries. The solid line at 0 signifies identical allele frequencies, indicating that at this value, genetic segments from both European and Indigenous ancestries exhibit equal allele frequencies. Dotted lines mark the average value for each database. All mean values are negative, demonstrating a predominant enrichment towards European Ancestry, which suggests that, on average, European segments have higher allele frequencies than Indigenous segments.

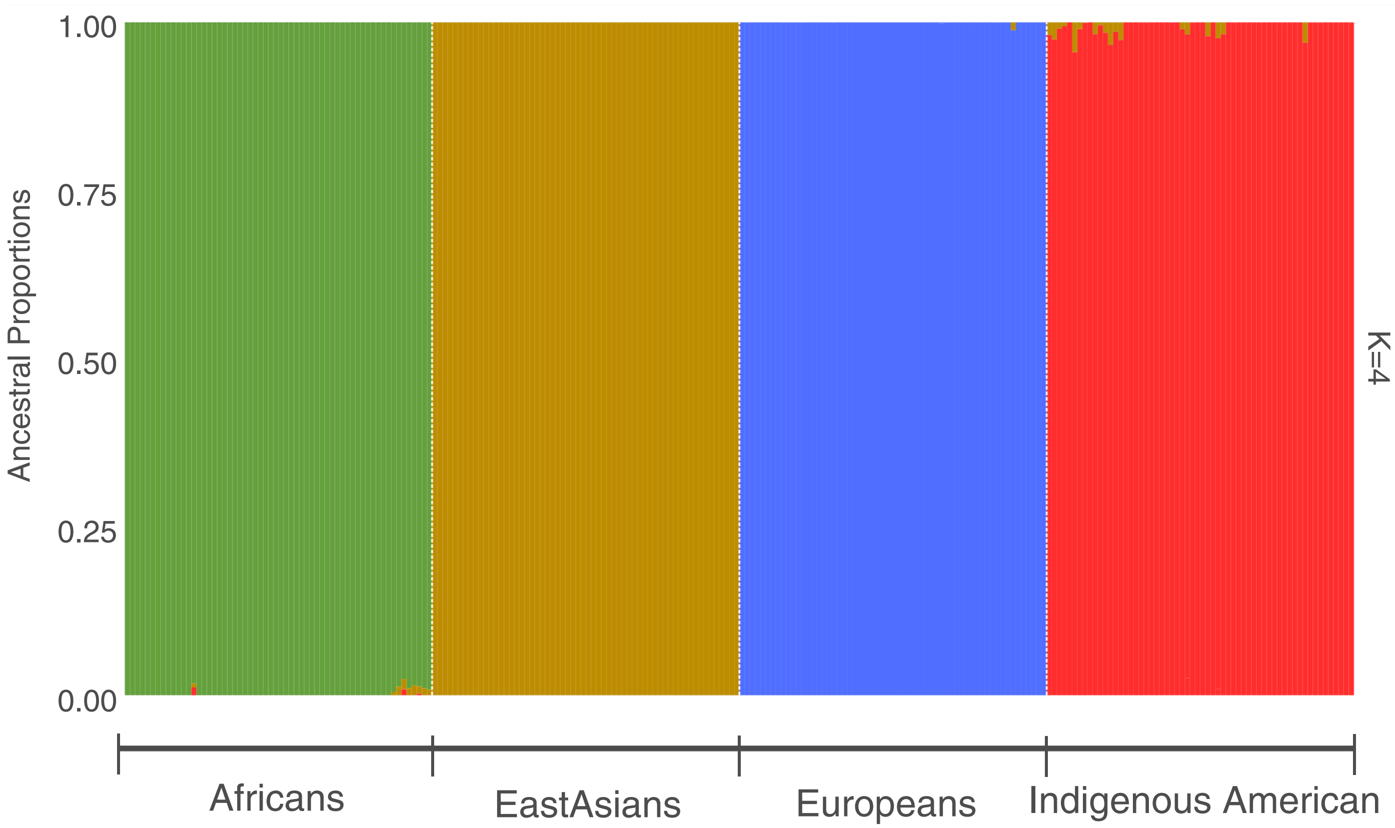

Supplementary Figure 14. Admixture Plot of Continental References used for Local Ancestry Calls

Admixture plot of continental references, unsupervised admixture run with K=4 . Reference populations were selected to represent the major genetic ancestries in Mexico: African (Afr), East Asian (Eas) , European (Eur), and Indigenous from the Americas (Ind). Individuals are plotted along the horizontal axis with their genetic ancestry proportions plotted as bars along the vertical.

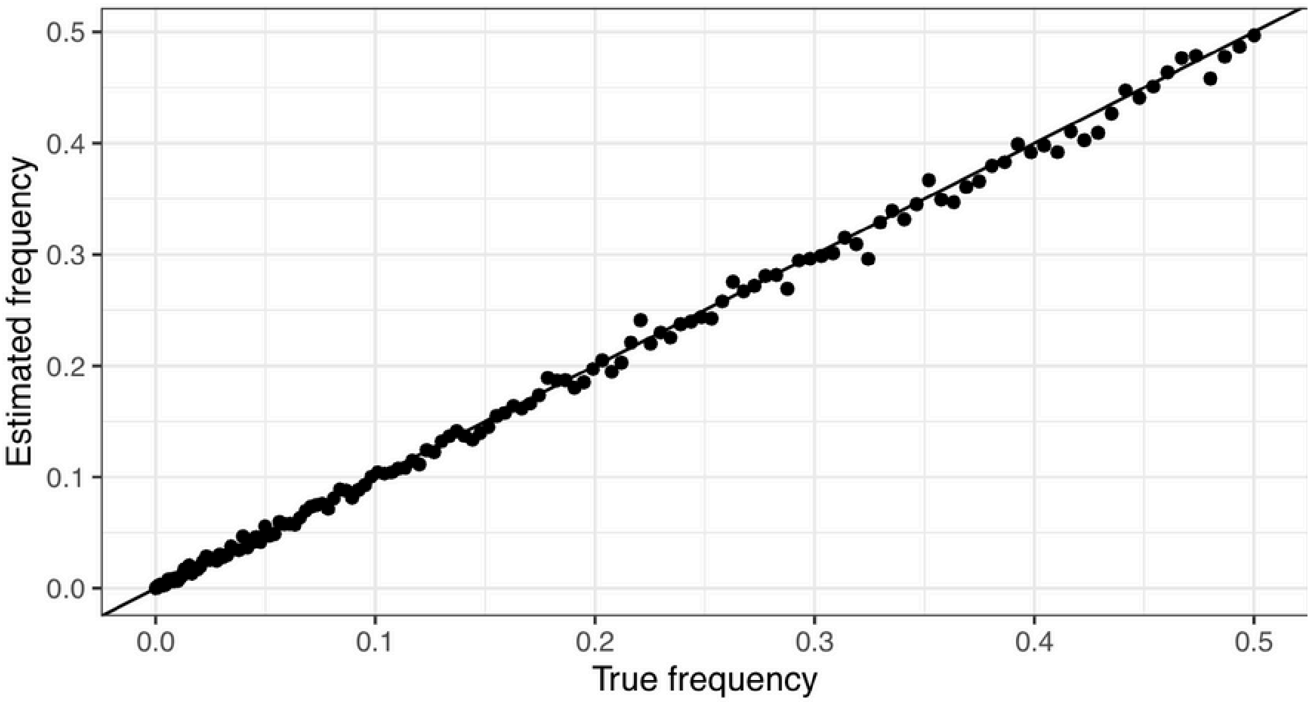

Supplementary Figure 15. Simulation of Local Ancestry Inference Error Impact on Allele Frequency Estimation.
Scatterplot comparing the true versus estimated ancestry-specific allele frequencies obtained from a simulation reflecting ancestry proportions in the Mexican population (AFR = 4%, AME = 65%, EUR = 30%, EAS = 1%). Frequencies were assigned per ancestry and local ancestry was predicted using a confusion matrix based on G-Nomix performance. Despite incorporating local ancestry misclassification, the estimated frequencies closely matched the true values across a range of allele frequencies, demonstrating the robustness of ancestry-specific frequency estimation in the presence of local ancestry inference errors.

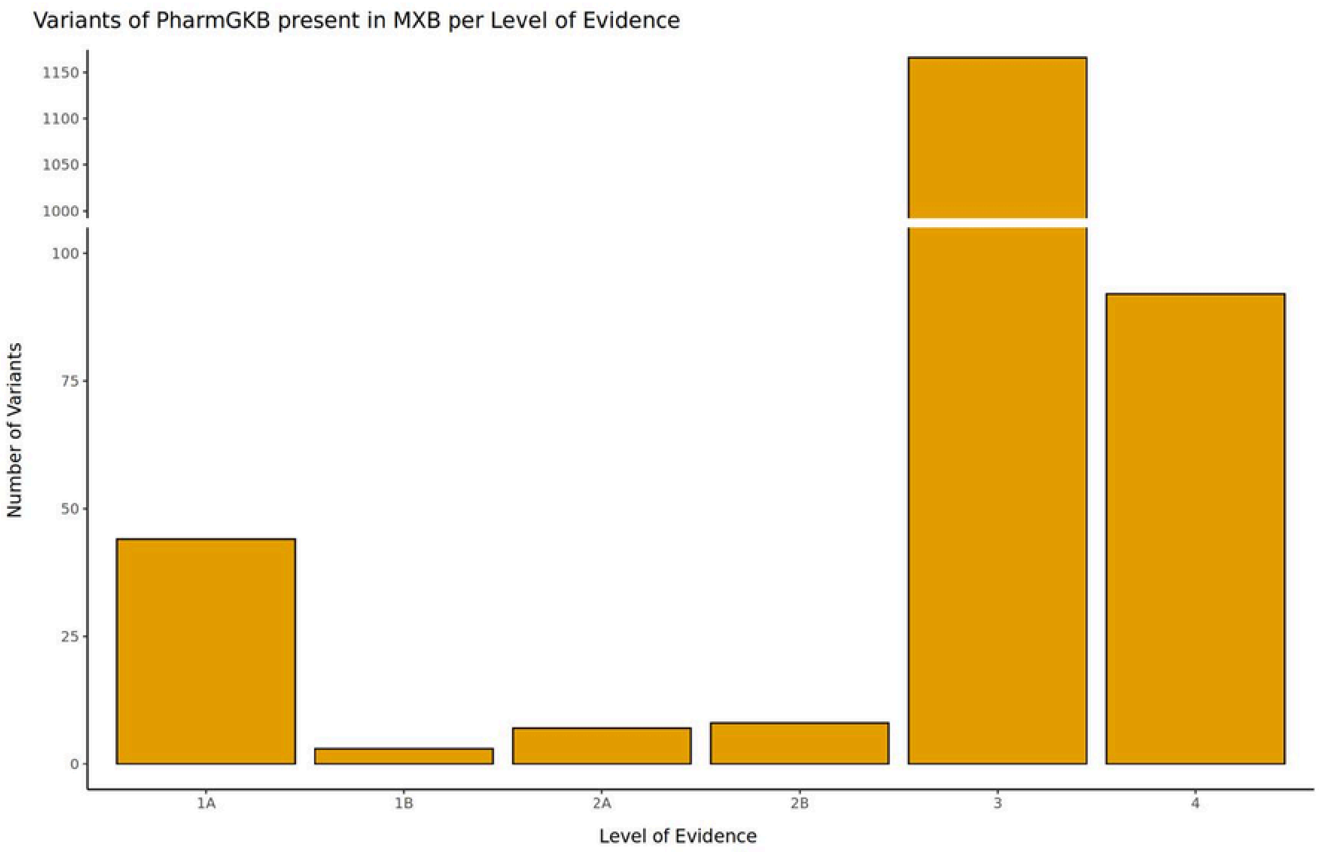

Supplementary Figure 16 . Distribution of PharmGKB Variants Present in the Mexican Biobank (MXB) by Level of Evidence.

Bar plot showing the number of pharmacogenomic variants from PharmGKB identified in the Mexican Biobank (MXB), categorized by PharmGKB's clinical annotation levels of evidence. A variant can be found in more that one category.
