# Supplementary material for: Clinical genetic variation across Hispanic populations in the Mexican Biobank
Source: Nat Med. 2026 Jan 21;32(2):725–35. doi: 10.1038/s41591-025-04100-z (PMC12920135; doi:10.1038/s41591-025-04100-z)
Supplement: Supplementary file 2 — Reporting Summary [file 41591_2025_4100_MOESM2_ESM.pdf]

nature portfolio

Corresponding author(s): Andres Moreno-Estrada

Last updated by author(s): Dec 2, 2025

Reporting Summary

Nature Portfolio wishes to improve the reproducibility of the work that we publish. This form provides structure for consistency and transparency in reporting. For further information on Nature Portfolio policies, see our Editorial Policies and the Editorial Policy Checklist.

Statistics

For all statistical analyses, confirm that the following items are present in the figure legend, table legend, main text, or Methods section.

n/a	Confirmed	
☐	☒	The exact sample size (*n*) for each experimental group/condition, given as a discrete number and unit of measurement
☒	☐	A statement on whether measurements were taken from distinct samples or whether the same sample was measured repeatedly
☐	☒	The statistical test(s) used AND whether they are one- or two-sided<br>*Only common tests should be described solely by name; describe more complex techniques in the Methods section.*
☐	☒	A description of all covariates tested
☒	☐	A description of any assumptions or corrections, such as tests of normality and adjustment for multiple comparisons
☐	☒	A full description of the statistical parameters including central tendency (e.g. means) or other basic estimates (e.g. regression coefficient) AND variation (e.g. standard deviation) or associated estimates of uncertainty (e.g. confidence intervals)
☐	☒	For null hypothesis testing, the test statistic (e.g. *F*, *t*, *r*) with confidence intervals, effect sizes, degrees of freedom and *P* value noted<br>*Give P values as exact values whenever suitable.*
☒	☐	For Bayesian analysis, information on the choice of priors and Markov chain Monte Carlo settings
☒	☐	For hierarchical and complex designs, identification of the appropriate level for tests and full reporting of outcomes
☒	☐	Estimates of effect sizes (e.g. Cohen's *d*, Pearson's *r*), indicating how they were calculated

Our web collection on statistics for biologists contains articles on many of the points above.

Software and code

Policy information about availability of computer code

Data collection

The variant datasets used in the MexVar analysis were obtained from the following sources directly from there websites by the end of March 2023:

ClinVar: https://www.ncbi.nlm.nih.gov/clinvar/
GWAS Catalog: https://www.ebi.ac.uk/gwas/
PharmGKB: https://www.pharmgkb.org/
Online Mendelian Inheritance in Man (OMIM): https://omim.org/

Data analysis

PLINK (v1.9): Used for quality control, genotype pruning, Fst calculation, and SNP filtering.
ADMIXTURE (v1.3.0): Applied for global ancestry inference.
VCFtools: Used for allele frequency calculations.
Bcftools (v1.13): Performed allele frequency calculations and variant filtering.
Gnomix: Utilized for local ancestry inference (GitHub repository).
Shiny Application (R framework) for MexVar:
Developed the MexVar app for real-time allele frequency exploration, incorporating the following libraries:
Core Libraries: shiny, shinydashboard, data.table, dplyr, mxmaps, colourpicker, stringr, ggplot2, ggpubr, gridExtra, esquisse, viridis, scales, DT, tidyr
Additional R-based tools used outside of the Shiny app:
Visualization & Data Wrangling: ggplot2, ggrepel, scales, mxmaps, dplyr, stringr, readr, MetBrewer, pheatmap
Custom Scripts for Local Ancestry-Specific Allele Frequencies (asF):
A custom Python (V3.6) script (available on GitHub https://github.com/morenolab/MexVar_Paper_Code) was developed to calculate

ancestry-specific allele frequencies using the following libraries:
Python Libraries: pandas, numpy, cyvcf2, allel

For manuscripts utilizing custom algorithms or software that are central to the research but not yet described in published literature, software must be made available to editors and reviewers. We strongly encourage code deposition in a community repository (e.g. GitHub). See the Nature Portfolio guidelines for submitting code & software for further information.

Data

Policy information about availability of data

All manuscripts must include a data availability statement. This statement should provide the following information, where applicable:

- Accession codes, unique identifiers, or web links for publicly available datasets
- A description of any restrictions on data availability
- For clinical datasets or third party data, please ensure that the statement adheres to our policy

Individual level genotype data were previously generated as part of The Mexican Biobank Project (MXB) , and are available at the European Genome-phenome Archive (EGA) through a Data Access Agreement with the Data Access Committee (EGA accession number for study: EGAS00001005797; dataset: EGAD00010002361 (Mexican_Biobank_Genotypes) Additionally, MXB frequency data have been uploaded as a new track to the UCSC human genome browser, and ancestry-specific frequencies are available through MexVar at https://morenolab.shinyapps.io/mexvar/.

The variant datasets used in the MexVar analyses were obtained from the following sources by the end of March 2023:

ClinVar: https://www.ncbi.nlm.nih.gov/clinvar/
GWAS Catalog: https://www.ebi.ac.uk/gwas/
PharmGKB: https://www.pharmgkb.org/
Online Mendelian Inheritance in Man (OMIM): https://omim.org/

Research involving human participants, their data, or biological material

Policy information about studies with human participants or human data. See also policy information about sex, gender (identity/presentation), and sexual orientation and race, ethnicity and racism.

Reporting on sex and gender	This work did not involve recruitment of human participants. Demographics of the human subjects included in the MXB study are described in Sohail et al., Nature, 2023.
Reporting on race, ethnicity, or other socially relevant groupings	This work did not involve recruitment of human participants. Demographics of the human subjects included in the MXB study are described in Sohail et al., Nature, 2023.
Population characteristics	This work did not involve recruitment of human participants. Demographics of the human subjects included in the MXB study are described in Sohail et al., Nature, 2023.
Recruitment	This work did not involve recruitment of human participants. Demographics of the human subjects included in the MXB study are described in Sohail et al., Nature, 2023.
Ethics oversight	This work did not involve recruitment of human participants. Demographics of the human subjects included in the MXB study are described in Sohail et al., Nature, 2023. Ethics approval was obtain from the Research Ethics Committee of the National Institute of Public Health in Mexico (IRB approvals CI: 1479 and CB: 1470)

Note that full information on the approval of the study protocol must also be provided in the manuscript.

Field-specific reporting

Please select the one below that is the best fit for your research. If you are not sure, read the appropriate sections before making your selection.

☒ Life sciences ☐ Behavioural & social sciences ☐ Ecological, evolutionary & environmental sciences

For a reference copy of the document with all sections, see nature.com/documents/nr-reporting-summary-flat.pdf

Life sciences study design

All studies must disclose on these points even when the disclosure is negative.

Sample size	No statistical methods were used to predetermine sample size. The sample size was selected before analysis was begun based on available samples and budgetary constraints for genotyping. We sought to include sufficient sample to power statistical comparisons.
Data exclusions	We used plink to remove all individuals with more than 5% missing genotype data, and all genotypes with more than 5% missing individuals. We restricted the analyses to only autosomes and removed all monomorphic SNPs. We restricted the analysis to only biallelic SNPs and

removed all SNPs with ambiguous strand for all downstream analyses using SNPFLIP. All related individuals were detected using plink (--Z-genome --min 0.5) after pruning for LD (--indep-pairwise 50 5 0.5).

Replication	No experiments were conducted, so replication of experimental results is not relevant.
Randomization	This is not relevant as our study does not consider variable assignments or categories.
Blinding	Blinding was not relevant to this study, as it is not a clinical trial or association study, but rather a descriptive population genetics analysis.

Reporting for specific materials, systems and methods

We require information from authors about some types of materials, experimental systems and methods used in many studies. Here, indicate whether each material, system or method listed is relevant to your study. If you are not sure if a list item applies to your research, read the appropriate section before selecting a response.

Materials & experimental systems

n/a	Involved in the study
☒	☐ Antibodies
☒	☐ Eukaryotic cell lines
☒	☐ Palaeontology and archaeology
☒	☐ Animals and other organisms
☒	☐ Clinical data
☒	☐ Dual use research of concern
☒	☐ Plants

Methods

n/a	Involved in the study
☒	☐ ChIP-seq
☒	☐ Flow cytometry
☒	☐ MRI-based neuroimaging

Plants

Seed stocks	*Report on the source of all seed stocks or other plant material used. If applicable, state the seed stock centre and catalogue number. If plant specimens were collected from the field, describe the collection location, date and sampling procedures.*
Novel plant genotypes	*Describe the methods by which all novel plant genotypes were produced. This includes those generated by transgenic approaches, gene editing, chemical/radiation-based mutagenesis and hybridization. For transgenic lines, describe the transformation method, the number of independent lines analyzed and the generation upon which experiments were performed. For gene-edited lines, describe the editor used, the endogenous sequence targeted for editing, the targeting guide RNA sequence (if applicable) and how the editor was applied.*
Authentication	*Describe any authentication procedures for each seed stock used or novel genotype generated. Describe any experiments used to assess the effect of a mutation and, where applicable, how potential secondary effects (e.g. second site T-DNA insertions, mosiacism, off-target gene editing) were examined.*
